# The long non-coding RNA *HOXB-AS3* regulates ribosomal RNA transcription in *NPM1*-mutated acute myeloid leukemia

**DOI:** 10.1038/s41467-019-13259-2

**Published:** 2019-11-25

**Authors:** Dimitrios Papaioannou, Andreas Petri, Oliver M. Dovey, Sara Terreri, Eric Wang, Frances A. Collins, Lauren A. Woodward, Allison E. Walker, Deedra Nicolet, Felice Pepe, Prasanthi Kumchala, Marius Bill, Christopher J. Walker, Malith Karunasiri, Krzysztof Mrózek, Miranda L. Gardner, Virginia Camilotto, Nina Zitzer, Jonathan L. Cooper, Xiongwei Cai, Xiaoqing Rong-Mullins, Jessica Kohlschmidt, Kellie J. Archer, Michael A. Freitas, Yi Zheng, Robert J. Lee, Iannis Aifantis, George Vassiliou, Guramrit Singh, Sakari Kauppinen, Clara D. Bloomfield, Adrienne M. Dorrance, Ramiro Garzon

**Affiliations:** 1 The Ohio State University, Comprehensive Cancer Center, Columbus, OH USA; 2 Center for RNA Medicine, Department of Clinical Medicine, Aalborg University, Copenhagen, Denmark; 3 Wellcome Trust Sanger Institute, Wellcome Trust Genome Campus, Cambridge, UK; 4 Institute of Genetics and Biophysics (IGB-ABT), National Council of Research (CNR), Naples, Italy; 5 Department of Pathology, New York University School of Medicine, New York, NY USA; 6 Department of Molecular Genetics, Center for RNA Biology, The Ohio State University, Columbus, OH USA; 7 Alliance for Clinical Trials in Oncology Statistics and Data Center, The Ohio State University, Columbus, OH USA; 8 Department of Cancer Biology and Genetics, The Ohio State University, Columbus, OH USA; 9 Department of Molecular Medicine, University of Pavia, Pavia, Italy; 10 Cancer and Blood Diseases Institute, Cincinnati Children’s Hospital Medical Center, University of Cincinnati, Cincinnati, OH USA; 11 Division of Biostatistics, College of Public Health, The Ohio State University, Columbus, OH USA; 12 Division of Pharmaceutics, College of Pharmacy, The Ohio State University, Columbus, OH USA; 13 Department of Haematology, Cambridge University Hospitals NHS Trust, Cambridge, UK

**Keywords:** Cancer, Molecular biology, Non-coding RNAs, Transcription, Molecular medicine

## Abstract

Long non-coding RNAs (lncRNAs) are important regulatory molecules that are implicated in cellular physiology and pathology. In this work, we dissect the functional role of the *HOXB-AS3* lncRNA in patients with *NPM1*-mutated (*NPM1*mut) acute myeloid leukemia (AML). We show that *HOXB-AS3* regulates the proliferative capacity of *NPM1*mut AML blasts in vitro and in vivo. *HOXB-AS3* is shown to interact with the ErbB3-binding protein 1 (EBP1) and guide EBP1 to the ribosomal DNA locus. Via this mechanism, *HOXB-AS3* regulates ribosomal RNA transcription and de novo protein synthesis. We propose that in the context of *NPM1* mutations, *HOXB-AS3* overexpression acts as a compensatory mechanism, which allows adequate protein production in leukemic blasts.

## Abstract (web summary)

LncRNAs at the *HOX* gene locus are increased in expression in *NPM1* mutant acute myeloid leukemia. In this study, the authors show that one such lncRNA, HOX3B-AS3, has a role in regulating gene transcription and protein synthesis in leukemia cells.

## Introduction

Acute myeloid leukemia (AML) is a highly heterogeneous disease with regard to its underlying genetic abnormalities and clinical course^1^. Recurrent chromosome aberrations and gene mutations have been implicated in leukemogenesis and are used in the clinic to risk-stratify treatment of AML patients^2^. Mutations in the *NPM1* gene are among the most frequent and clinically relevant genetic alterations in AML, and define a distinct subtype of AML in the World Health Organization classification of acute leukemias^3^. *NPM1* mutations are detected in ~60% of patients with cytogenetically normal AML (CN-AML) and associate with a favorable clinical outcome^4–6^.

The wild-type *NPM1* gene encodes an ubiquitously expressed protein that shows strong nucleolar localization^7^, shuttles between the nucleus and cytoplasm^8^ and regulates important cellular functions such as transcription^9^ and maturation^10,11^ of ribosomal RNA (rRNA) and nuclear export of ribosomal proteins^12^. *NPM1* mutations in AML result in the introduction of a nuclear export signal at the C-terminus of the NPM1 protein, which leads to the aberrant localization of the mutant protein in the cytoplasm^4,13,14^. At the molecular level, *NPM1*-mutated CN-AML is associated with a distinctive mRNA-expression profile characterized by *HOX* gene family overexpression and CD34 negativity^6,15^. Mechanistically, expression of a humanized mutant *Npm1* allele in a conditional knock-in mouse model causes *HOX* gene overexpression and late-onset myeloid leukemia^16^. These findings support the critical role of *NPM1* mutations in leukemogenesis.

Long non-coding RNAs (lncRNAs) constitute a distinct subset of non-protein-coding RNA molecules that are longer than 200 nucleotides and regulate many key cellular functions^17^. Deregulated expression of lncRNAs is an important molecular event in many types of cancer^18–23^. In AML, prognostic lncRNA transcripts whose expression independently associates with the outcome of AML patients have recently been identified^24–26^. In addition, recurrent prognostic gene mutations in AML, including *NPM1* mutations, have been reported to associate with distinctive lncRNA signatures^24,25,27^. A lncRNA embedded in the *HOXB* locus and named *HOXB-AS3* was identified among the most highly upregulated lncRNAs in CN-AML patients with *NPM1* mutations.

*HOX* genes are important regulators of embryonic and hematopoietic cell development, which have been implicated in leukemogenesis^28^. *HOX* gene loci also contain lncRNAs that are involved in cell differentiation and cancer pathogenesis (e.g., *HOTAIR*, *HOTTIP*, *HOTAIRM1*), mainly by regulating the expression levels of protein-coding *HOX* genes^29–31^.

Considering the aberrant expression of the *HOX* genes in AML with mutated *NPM1* (*NPM1*mut) and the role of lncRNAs in regulating *HOX* gene expression, we hypothesized that *HOXB-AS3* overexpression could have biologic significance in *NPM1*mut AML. Herein, we dissect the functional role of *HOXB-AS3* in *NPM1*mut AML. We perform assays to identify the protein interactors of *HOXB-AS3* and describe its functional role in regulating rRNA transcription and ribosomal biogenesis in leukemic blasts. Finally, we demonstrate the potential value of *HOXB-AS3* as a therapeutic target in preclinical AML xenograft models.

## Results

### *HOXB-AS3* expression in healthy hematopoiesis and AML

**Fig. 1:**
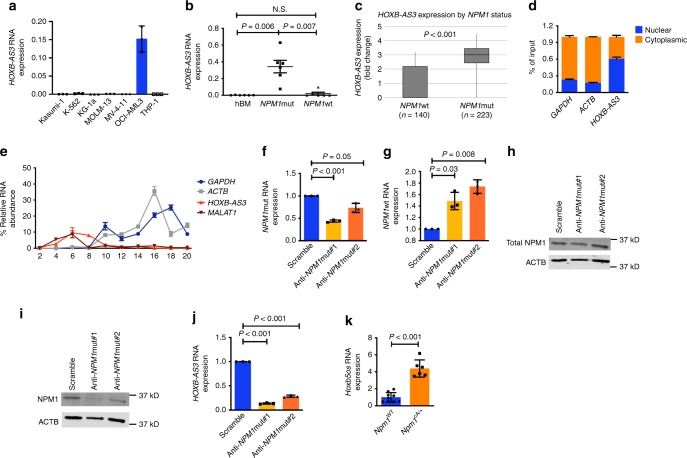
*HOXB-AS3* lncRNA expression in healthy hematopoietic cells and leukemic blasts. **a** Relative RNA expression of *HOXB-AS3* in seven AML cell lines. **b** Relative *HOXB-AS3* RNA expression in bone marrow (BM) samples from six healthy donors (hBM), AML blasts from six patients with *NPM1*mut, and AML blasts from six patients with *NPM1*wt, in aggregate. N.S., not significant. **c** *HOXB-AS3* RNA expression (depicted as fold change) in younger adult CN-AML patients with *NPM1* wild-type (*NPM1*wt; *n* = 140) and in those with mutated *NPM1* (*NPM1*mut; *n* = 223). **d** Nuclear versus cytoplasmic localization of *GAPDH*, *ACTB* mRNA, and *HOXB-AS3* RNA in OCI-AML3 cells. **e** Targeted profiling of *GAPDH*, *ACTB*, *HOXB-AS3*, and *MALAT1* in OCI-AML3 cells in non-polysome associated (fractions 1–6), 40S, 60S, 80S (fractions 7–11), as well as low (fractions 12–15) and high (fractions 16–20) molecular weight polysomal fractions, isolated via glucose gradient centrifugation. **f**–**k** OCI-AML3 cells treated with scramble versus *NPM1*mut-targeting gapmers (anti-*NPM1*mut#1 and anti-*NPM1*mut#2). **f** Relative *NPM1*mut mRNA, **g** relative *NPM1*wt mRNA, **h** total NPM1 protein, **i** NPM1mut protein, and **j** relative *HOXB-AS3* RNA expression in the treated cells. In the presented western blots (**h**, **i**), ACTB is also visualized as loading control. **k** Relative *Hoxb5os* RNA expression in BM samples from three *Npm1*^WT^ and two *Npm1*^cA/+^ mice. Unless otherwise stated all samples were analyzed in technical triplicates. *P* values were calculated using paired two-sided *t*-tests. In the figures, heights of boxplots indicate mean values with standard deviation. Error bars indicate highest and lowest values in each population. Source data are provided as a Source Data file.

We first measured the expression levels of *HOXB-AS3* in six AML cell lines with wild-type *NPM1* (*NPM1*wt) (Kasumi-1, K-562, KG-1a, MOLM-13, MV-4-11, and THP-1) and in the *NPM1*mut OCI-AML3 cell line, which harbors Type A *NPM1* mutations, by custom-designed real-time quantitative PCR (RT-qPCR) (Supplementary Table 1a). We focused on the *HOXB-AS3* transcript variant NR_033202.2, which was found to be dominantly expressed in AML blasts (Supplementary Fig. 1). We found detectable levels of *HOXB-AS3* only in the *NPM1*mut OCI-AML3 cells (Fig. 1a).

We further analyzed bone marrow (BM) samples from healthy donors as well as leukapheresis samples from *NPM1*mut and *NPM1*wt AML patients (*n* = 6 in each group) using RT-qPCR. Cytogenetic and mutational profiles of the AML patients are provided in Supplementary Table 2. We found that *NPM1*mut patients had higher *HOXB-AS3* expression than *NPM1*wt patients (*P* = 0.007) or healthy BM cells (*P* = 0.006; Fig. 1b). There was no significant difference in *HOXB-AS3* expression between healthy BM cells and the *NPM1*wt AML samples. We further examined the impact of *NPM1*mut status on *HOXB-AS3* expression in publicly available data of AML patients previously analyzed with RNA-seq^25^. *NPM1*mut patients (*n* = 223) had approximately a three-fold increase in *HOXB-AS3* expression compared with *NPM1*wt patients (*n* = 140; Fig. 1c, *P* *<* 0.001). *HOXB-AS3* upregulation was observed independently of the type of *NPM1* mutations (Supplementary Fig 2a, b). Last, we queried datasets of AML samples and normal hematopoietic cells analyzed with microarray assays or RNA-seq and deposited in the BloodSpot portal (www.bloodspot.eu). In keeping with our findings, *HOXB-AS3* was not expressed in healthy BM cells and was detected in CN-AML samples and, in particular, in *NPM1*mut CN-AML (Supplementary Fig. 3a, c).

### *HOXB-AS3* does not associate with polysomes in AML cells

To gain insights into the functional role of *HOXB-AS3*, we first studied its subcellular localization. We purified nuclear and cytoplasmic fractions of OCI-AML3 cells and measured the abundance of the *GAPDH* mRNA, *ACTB* mRNA, and *HOXB-AS3* lncRNA in these subcellular compartments. In keeping with their protein-coding function, the *GAPDH* and *ACTB* transcripts were primarily located in the cytoplasm of OCI-AML3 cells. In contrast, *HOXB-AS3* was more abundant in the nuclear fraction of the cells (Fig. 1d). To determine whether *HOXB-AS3* has any protein-coding potential, we used sucrose gradient-based fractionation and performed targeted polysome profiling in OCI-AML3 cells. We measured the abundance of *GAPDH* mRNA, *ACTB* mRNA, *HOXB-AS3* lncRNA, and *MALAT1* lncRNA, which is an established non-coding transcript^32^, on the isolated fractions. Both *GAPDH* and *ACTB* were enriched in the low and high molecular weight polysomal fractions, whereas *HOXB-AS3* and *MALAT1*, were enriched in the free fractions, indicating that these transcripts do not interact with the translational machinery of the blasts (Fig. 1e).

### *HOXB-AS3* overexpression is driven by *NPM1* mutations

To examine the association between *NPM1* mutations and *HOXB-AS3* overexpression, we performed knockdown (KD) experiments of the *NPM1*mut allele in OCI-AML3 cells using *NPM1*mut-targeting locked nucleic acid (LNA)-modified gapmer antisense oligonucleotides (hereafter named gapmers). Electroporation-mediated delivery of either anti-*NPM1*mut gapmer (anti-*NPM1*mut#1 and anti-*NPM1*mut#2, Supplementary Table 1b) led to KD of *NPM1*mut (Fig. 1f) and a concomitant increase in the *NPM1*wt mRNA expression (Fig. 1g). There was no reduction in the amount of total NPM1 protein (Fig. 1h), while the amount of mutant NPM1 protein was decreased (Fig. 1i). At 48 h post electroporation, KD of *NPM*1mut led to a decrease in *HOXB-AS3* RNA abundance (*P* < 0.001; Fig. 1j).

We also analyzed lineage marker-negative, Kit-positive-selected murine BM cells, harvested from transgenic mice harboring a humanized, mutated *Npm1* knock-in allele (*Npm1*^cA/+^) and age-matched wild-type controls (*Npm1*^WT^), as previously described^16^. *Npm1*^cA/+^ mice harbor a four-nucleotide insertion that corresponds to the Type A *NPM1* mutations that are detected in human AML samples. We measured the expression levels of a murine lncRNA transcript, which is antisense to the *Hoxb5* and *Hoxb6* genes and displays sequence homology to the human *HOXB-AS3*, named *Hoxb5os*. We found *Hoxb5os* to be significantly upregulated in the *Npm1*^cA/+^ mice, compared with the *Npm1*^WT^ controls (*P* < 0.001; Fig. 1k).

### *HOXB-AS3* expression regulates proliferation of AML cells

**Fig. 2:**
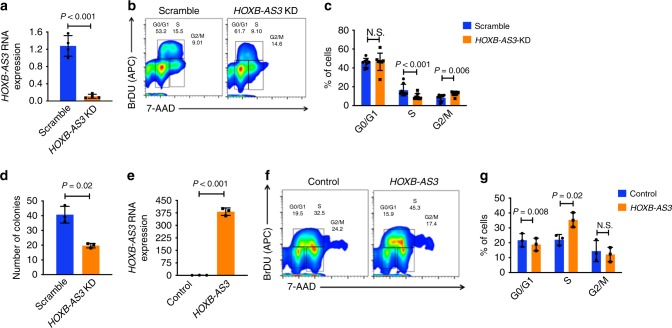
The *HOXB-AS3* lncRNA regulates cell proliferation of AML cells. **a** Relative *HOXB-AS3* RNA expression in OCI-AML3 cells treated with scramble versus anti-*HOXB-AS3* gapmers (*HOXB-AS3* KD). **b** Cell-cycle analysis in scramble versus anti-*HOXB-AS3* gapmer-treated OCI-AML3 cells. Results of a representative experiment are shown. **c** Comparison of the percentages of cells at each stage of the cell cycle (G0/G1, S, and G2/M phases) in OCI-AML3 cells treated with scramble versus anti-*HOXB-AS3* gapmers. Results of six independent experiments are shown. **d** Comparison of the numbers of colonies formed by scramble versus anti-*HOXB-AS3* gapmer-treated OCI-AML3 cells in colony-forming unit assays. Results of three independent experiments are shown. **e** Relative *HOXB-AS3* RNA expression in K-562 cells transfected with empty vector (control) versus a *HOXB-AS3*-overexpressing vector. **f** Cell-cycle in K-562 cells transfected with control versus *HOXB-AS3*-overexpressing vectors. Results of a representative experiment are shown. **g** Comparison of the percentages of cells at each stage of the cell cycle (G0/G1, S, and G2/M phases) in K-562 cells transfected with control versus a *HOXB-AS3*-overexpressing vectors. Results of three independent experiments are shown. *P* values were calculated using paired two-sided *t*-tests. In the figures, heights of boxplots indicate mean values with standard deviation. Error bars indicate highest and lowest values in each population. Source data are provided as a Source Data file.

Next, we conducted KD experiments using anti-*HOXB-AS3* gapmers. Delivery of a mixture of two gapmers (anti-*HOXB-AS3*#1 and anti-*HOXB-AS3*#2, Supplementary Table 1b) to OCI-AML3 cells led to a 10-fold decrease of *HOXB-AS3* expression (*P* = 0.003; Fig. 2a). Cell-cycle analysis based on bromodeoxyuridine (BrdU) incorporation and 7-actinomycin D (7-AAD) staining, conducted at 48 h post-delivery of gapmers, showed a decrease in the fraction of OCI-AML3 cells in S phase upon KD of *HOXB-AS3* (*P* < 0.001) and an increase in the G2/M phase cells compared with scramble control (*P* = 0.006*;* Fig. 2b, c). Annexin V/propidium iodide staining analysis showed no difference in apoptosis between scramble and anti-*HOXB-AS3*-treated cells (Supplementary Fig. 4a, b). Colony formation unit (CFU) assays in methylcellulose-based media showed a decrease in the colony-forming capacity of OCI-AML3 cells after *HOXB-AS3* KD (*P* = 0.02; Fig. 2d).

In contrast, overexpression of *HOXB-*AS3 in K-562 cells (*P* = 0.001; Fig. 2e) led to an increase in the fraction of the proliferating blasts (*P* = 0.02; Figs. 2f and g), with a concomitant decrease in the fraction of cells in the G0/G1 phase (*P* = 0.008). Taken together, these data indicate the regulatory effect of *HOXB-AS3* expression levels on the proliferative capacity of leukemic blasts.

### *HOXB-AS3* KD decreases proliferation of AML patient blasts

**Fig. 3:**
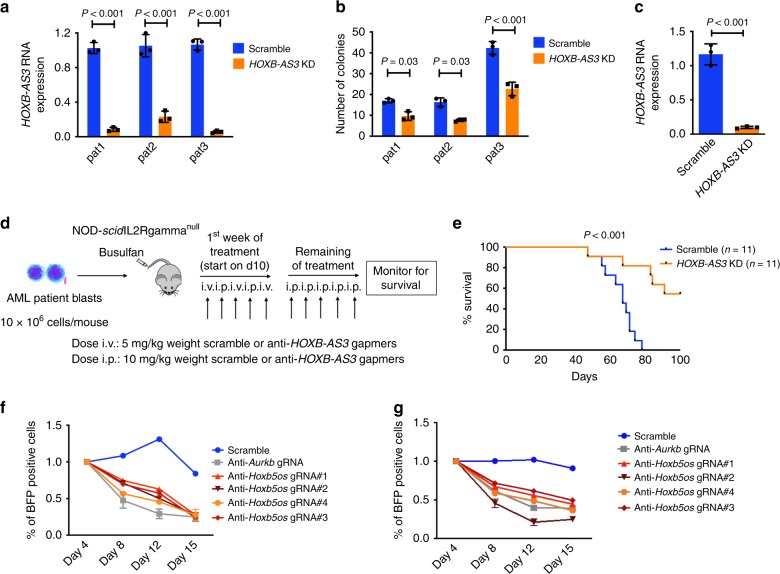
Functional significance of *HOXB-AS3* expression in AML patient blasts. **a** Relative *HOXB-AS3* RNA expression in AML blasts from three patients with *NPM1* mutations (*NPM1*mut), treated with scramble versus anti-*HOXB-AS3* gapmers (*HOXB-AS3* KD). **b** Number of colonies formed by scramble versus *HOXB-AS3* KD AML patient blasts in colony-forming unit assays. Experiments were conducted in triplicates. **c** Relative *HOXB-AS3* RNA expression in AML blasts from a *NPM1*mut AML patient treated in vivo with scramble or anti-*HOXB-AS3* gapmers. **d** Schematic representation of the study design testing therapeutic value of in vivo *HOXB-AS3* KD. **e** Kaplan–Meier curves depicting survival of NSG mice xeno-transplanted with blasts of *NPM1*mut AML patients treated with scramble (*n* = 11) or anti-*HOXB-AS3* gapmers (*n* = 11). **f**, **g** Frequency of BFP-expressing cells transduced with either anti-*Hoxb5os* gRNAs or non-targeting controls over a 15-day period of competitive co-culture with non-transduced cells of the **f** *Rosa26*^Cas9/+^;*Npm1*^cA/+^*;Flt3*^ITD/+^ or the **g** *Rosa26*^Cas9/+^;*Npm1*^cA/+^;*Nras*^G12D/+^ murine AML cell lines. gRNAs targeting the *Aurkb* gene were used as a pan-essential positive controls. In (**a**–**c**), *P* values were calculated using paired two-sided *t*-tests. Heights of boxplots indicate mean values with standard deviation. Error bars indicate highest and lowest values in each population. In (**e**–**g**), *P* values were calculated using the log-rank test. Source data are provided as a Source Data file.

We conducted *HOXB-AS3* KD experiments in blasts of three *NPM1*mut AML patients (pat1, pat2, and pat3). Delivery of anti-*HOXB-AS3* gapmers via electroporation effectively targeted and downregulated *HOXB-AS3* RNA expression in blasts of each patient (Fig. 3a). *HOXB-AS3* KD led to a significant decrease in the number of formed colonies in CFU assays, compared with scramble control (Fig. 3b). In contrast, transfection with anti-*HOXB-AS3* gapmers had no effect in the proliferation of leukemic blasts of *NPM1*wt patients or the growth and differentiation of healthy hematopoietic progenitor cells (CD34 + selected umbilical cord blood cells, Supplementary Fig. 5a–e).

### *HOXB-AS3* KD prolongs survival of xeno-transplanted mice

To evaluate the effect of *HOXB-AS3* KD in AML blasts in vivo, we conducted experiments in patient-derived xenograft (PDX) mouse models. We used published protocols^33^ to transplant busulfan-pre-treated NOD-scid IL2Rgamma^null^ (NSG) mice with leukemic blasts of a *NPM1*mut AML patient in two independent experiments. Nine mice engrafted with patient AML blasts were treated with either anti-*HOXB-AS3* gapmers (*n* = 5) or scramble control (*n* = 4) for 2 weeks for evaluation of the in vivo *HOXB-AS3* KD efficacy. Treatment with lipid nanoparticle (LNP)-formulated anti-*HOXB-AS3* gapmers led to downregulation of *HOXB-AS3* expression in human CD45-selected AML blasts isolated from the BM of the treated mice (*P* < 0.001; Fig. 3c). Larger cohorts of mice were then engrafted with blasts. Ten days after transplantation, mice were divided into anti-*HOXB-AS3* (*n* = 11) or scramble treatment arms (*n* = 11) and were monitored for survival. An overview of the experimental design is provided in Fig. 3d. In vivo *HOXB-AS3* KD led to significant prolongation of survival of the treated mice compared with the scramble control. The median survival of the scramble treated mice was 63 days, whereas it was not reached after 100 days of treatment for the *HOXB-AS3* KD group (*P* < 0.001; Fig. 3e).

### *Hoxb5os* KD decreases proliferation of murine AML blasts

To further validate our observations of the *HOXB-AS3* functional significance, we employed CRISPR-Cas9 disruption of the -murine synonymous- *Hoxb5os* transcript, in two recently developed mouse models of *Npm1*^cA/+^-driven AML^34^. Specifically, we transduced the *Rosa26*^Cas9/+^; *Npm1*^cA*/*+^; *Flt3*^ITD/*+*^ and the *Rosa26*^Cas9/+^; *Npm1*^cA/+^; *Nras*^G12D/+^ cell lines with four combinations of dual guide RNAs (gRNA1-gRNA4, Supplementary Table 1c), which targeted the *Hob5os* region and co-expressed blue fluorescent protein (BFP). Similar BFP-expressing empty vectors were used as negative controls, whereas *Aurkb* targeting gRNAs were used as pan-essential positive controls^34^. Transduced cells were then grown in competitive co-culture conditions with non-transduced cells. Disruption of *Hoxb5os* led to a gradual reduction in the BFP-expressing cells after 15 days of culture in both *Npm1*^cA/+^-harboring cell lines, while cells transduced with control vectors remained unaffected (Figs. 3f and g).

### *HOXB-AS3* does not affect *HOX* gene expression

Several *HOX* loci-embedded lncRNAs are important molecular players in cancer by regulating in cis or in trans expression levels of *HOX* genes^29–31^. To examine whether *HOXB-AS3* regulates *HOX* gene expression we performed RNA-seq analyses in scramble versus anti-*HOXB-AS3-*treated OCI-AML3 cells (12, 24, and 48 h) and in blasts of four *NPM1*mut AML patients (24 h post transfection). *HOXB-AS3* KD had no effect on the expression levels of the *HOX* genes that are detectably expressed in *NPM1*mut AML patient blasts (Supplementary Fig. 6a, b) or the OCI-AML3 cells (Supplementary Fig. 6c–g). We validated these findings for a subset of *HOX* transcripts in OCI-AML3 cells by RT-qPCR (Supplementary Fig. 6h–k). As *HOXA9* overexpression has leukemogenic potential^35^, we further assessed HOXA9 protein levels by western blotting (WB). No difference in HOXA9 levels was detected between OCI-AML3 cells treated with scramble or anti-*HOXB-AS3* gapmers (Supplementary Fig. 6l).

### The ErbB3-binding protein 1 (EBP1) interacts with *HOXB-AS3*

**Fig. 4:**
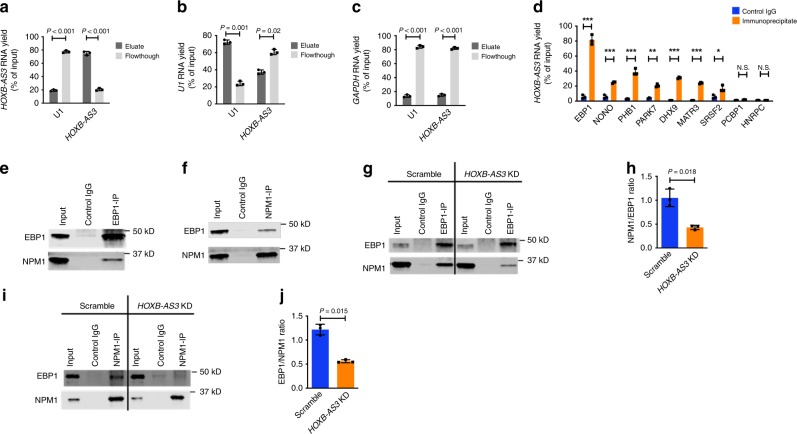
EBP1 strongly interacts with *HOXB-AS3* in *NPM1*mut AML cells. **a**–**c** Yields of **a** *HOXB-AS3* RNA, **b** *U1* RNA, and **c** *GAPDH* RNA in the eluates and the flow-through of lysates hybridized with *U1* and *HOXB-AS3*-targeting probes. The RNA yield is depicted as a percentage of the amount of the respective transcript in the input sample. **d** Validation of *HOXB-AS3*-protein interactions via RNA-Immunoprecipitation (RIP) experiments with nine candidate proteins. Enrichment of each immunoprecipitate is compared with the respective IgG control (mouse or rabbit). *P < 0.05, **P < 0.005, ***P < 0.001, N.S., not significant. **e** Immunoprecipitation of the EBP1 protein in nuclear lysates of OCI-AML3 cells followed by western blotting (WB) for the EBP1 and NPM1 proteins. **f** Immunoprecipitation of the NPM1 protein in nuclear lysates of OCI-AML3 cells followed by WB for the EBP1 and NPM1 proteins. **g**–**j** Effect of *HOXB-AS3* depletion (*HOXB-AS3* KD) on the formation of the EBP1-NPM1 complex in OCI-AML3 cells: **g** EBP1 immunoprecipitation in scramble versus *HOXB-AS3* KD-treated cells followed by WB for the EBP1 and NPM1 proteins. **h** Quantification of three independent experiments. **i** NPM1 immunoprecipitation in scramble versus *HOXB-AS3* KD-treated cells followed by WB for the EBP1 and NPM1 proteins. **j** Quantification of three independent experiments. *P* values were calculated using paired two-sided *t*-tests. In the figures, heights of boxplots indicate mean values with standard deviation. Error bars indicate highest and lowest values in each population. Source data are provided as a Source Data file.

Many lncRNAs regulate key cellular processes by interacting with specific proteins^29,36^. Thus, to further study the function of *HOXB-AS3*, we interrogated its interactions with the proteome of OCI-AML3 cells by performing RNA pull-down experiments and characterization of ribonucleoprotein complexes. To this end, we applied a modified version of the RNA-antisense purification technique^36^. In brief, we used biotinylated probes to capture either the *HOXB-AS3* lncRNA or the *U1* transcripts (Supplementary Table 1e) (Fig. 4a–c). Tandem mass spectrometry and comparative proteomic analyses of the *HOXB-AS3* and *U1* eluates were conducted and the putative *HOXB-AS3* and *U1* interactors are listed in Supplementary Table 3. Five proteins, reported to form the *U1*-complex (SNRNP70, SNRPE, SNRPD2, SNRPD3, and SNRPA)^37–39^ were identified as *U1*-interactors.

We also identified 22 putative *HOXB-AS3* interactors. Validation experiments by RNA immunoprecipitations (RIP) were conducted for nine of these 22 proteins and significant *HOXB-AS3* enrichment was found in seven of them (Fig. 4d). Of the tested candidates, EBP1 was the strongest interactor of *HOXB-AS3*, as it bound to approximately 80% of the measured *HOXB-AS3* in the input sample. We tested the specificity of the EBP1-*HOXB-AS3* interaction by profiling the RNA eluates that were isolated after EBP1 immunoprecipitations for other protein-coding and non-coding RNAs. In contrast to *HOXB-AS3*, none of the tested transcripts showed significant enrichment compared with controls, indicating that the interaction of EBP1 with *HOXB-AS3* is specific (Supplementary Fig. 7).

### *HOXB-AS3* regulates the interaction of EBP1 with NPM1

EBP1 is an RNA-binding protein first identified as an interactor of the ErbB3 receptor in human breast cancer cells. Two distinct EBP1 isoforms (p42 and p48) that differentially regulate proliferation and viability of cells^40^ have previously been identified. To elucidate if an EBP1 isoform preferentially interacts with *HOXB-AS3*, we cloned, labeled with a FLAG tag and overexpressed p42 and p48 in K-562 cells, which were co-transfected with *HOXB-AS3*-overexpressing vectors. RIP experiments showed that *HOXB-AS3* interaction with p48 was significantly stronger compared with the *HOXB-AS3*-EBP1p42 interaction (Supplementary Fig. 8).

Furthermore, the EBP1 protein has been reported to interact with NPM1 in the nucleolus and to regulate rRNA maturation^41^. EBP1 has also been shown to regulate transcription of rRNA species in AML cells^42^. Given this background, we examined the interaction of EBP1 and NPM1 proteins in the context of heterozygous *NPM1* mutations. We validated the EBP1-NPM1 interaction in nuclear lysates of OCI-AML3 cells by reciprocal co-immunoprecipitations (Figs. 4e and f). Similar experiments with a mutant NPM1-specific antibody showed that it is the wild-type NPM1 protein that primarily interacts with EBP1 (Supplementary Fig. 9). We next investigated how the *HOXB-AS3* levels affect the formation of the EBP1-NPM1 complex. *HOXB-AS3* KD in OCI-AML3 cells led to a decrease in the interacting amounts of EBP1 and NPM1 (Figs. 4g–j). *HOXB-AS3* KD had no effect in EBP1 mRNA or protein abundance in the transfected cells (Supplementary Fig. 10).

### The *HOXB-AS3-*EBP1-NPM1 complex regulates rRNA transcription

**Fig. 5:**
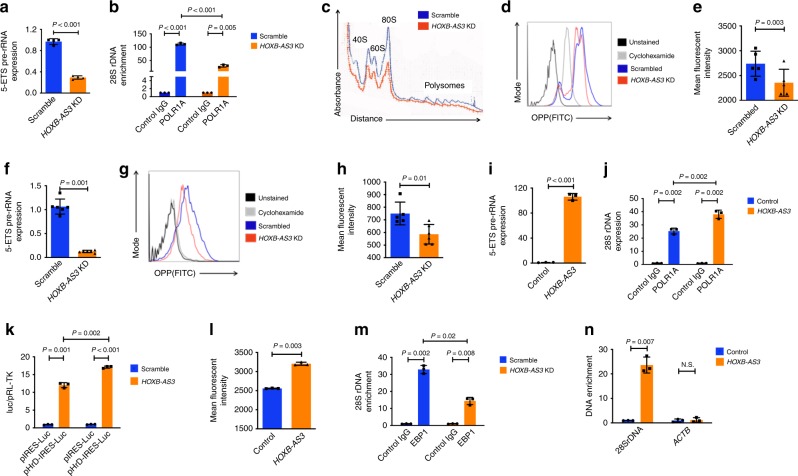
*HOXB-AS3* regulates rRNA transcription via guiding the EBP1 protein to the rDNA locus. **a** Relative pre-rRNA expression in OCI-AML3 cells treated with scramble or anti-*HOXB-AS3* gapmers. **b** Occupancy of ribosomal DNA promoter repeat sequence (28S rDNA) by POLR1A in scramble versus *HOXB-AS3* KD OCI-AML3 cells. **c** Sucrose gradient-based isolation and quantification (via ultraviolet radiation absorbance) of ribosomes and polysomes in scramble versus *HOXB-AS3* KD OCI-AML3 cells. **d** Effect of *HOXB-AS3* depletion on de novo protein synthesis in OCI-AML3 cells,The flow cytometry graph from one representative experiment is shown. **e** Comparison of the mean fluorescence intensity of the Alexa Fluor 488-positive OCI-AML3 cells treated with scramble versus *HOXB-AS3* KD, in de novo protein synthesis assays. Results of four independent experiments are shown. **f** Relative 5-ETS pre-rRNA expression in human AML patient blasts treated in vivo with scramble versus anti-*HOXB-AS3* gapmers. Results of three scramble and four *HOXB-AS3* KD-treated mice are depicted in aggregate. **g**, **h** Effect of *HOXB-AS3* KD on de novo protein synthesis in in vivo treated human AML blasts**. i** Relative pre-rRNA expression in K-562 cells transfected with empty vector (control) or *HOXB-AS3* overexpressing vectors. **j** Occupancy of 28S rDNA by POLR1A in K-562 cells transfected with control or *HOXB-AS3* overexpressing vectors. **k** Relative luciferase-reporter (luc/pRL-TK) activity in K-562 cells transfected with empty control (pIRES-Luc) or rDNA-promoter-containing (pHrD-IRES-Luc) luciferase-reporter vectors, and control or *HOXB-AS3* overexpressing vectors. **l** Comparison of the mean fluorescence intensity of the Alexa Fluor 488-positive K-562 cells, transfected with control or *HOXB-AS3* overexpressing vectors in de novo protein synthesis assays. Results of three independent experiments are shown. **m** Occupancy of 28S rDNA by EBP1 in scramble versus *HOXB-AS3* KD OCI-AML3 cells. **n** Enrichment of 28S rDNA or the *ACTB* promoter region in RAP-DNA-based isolation of chromatin with control or *HOXB-AS3*-hybridizing biotinylated probes*. P* values were calculated using paired two-sided *t*-tests. In the figures, heights of boxplots indicate mean values with standard deviation. Error bars indicate highest and lowest values in each population. Source data are provided as a Source Data file.

EBP1 has been shown to interact with NPM1 in the nucleus and independently regulate rRNA transcription in AML cells^41,42^. We therefore examined whether *HOXB-AS3* modulates the transcriptional activity at the ribosomal DNA (rDNA) locus in *NPM1*mut AML cells. We measured rRNA abundance as previously described^42^ and found that *HOXB-AS3* KD led to a decrease in the transcribed rRNA in OCI-AML3 cells (*P* < 0.001; Fig. 5a). In addition, *HOXB-AS3* KD led to a reduction in the occupancy of the rDNA-promoter by RNA Polymerase I (POLR1A) in chromatin-immunoprecipitation (ChIP) experiments (*P* < 0.001; Fig. 5b). Polysomal profiling revealed an overall reduction of the 40S and 60S ribosomal subunits as well as a reduction in the formed monosomes (80S) and polysomes upon *HOXB-AS3* KD (Fig. 5c). Consequently, in vitro labeling of the de novo synthesized polypeptides by O-proargyl-puromycin (OPP) indicated that *HOXB-AS3* KD led to a reduction in protein synthesis of OCI-AML3 cells, compared with scramble control (*P* = 0.003; Figs. 5d and e). Finally, short-term in vivo treatment of human AML blasts in murine xenografts revealed that *HOXB-AS3* KD led to a decrease in the amount of the transcribed rRNA species (*P* < 0.001; Fig. 5f) and a decrease in the de novo protein synthesis in these cells (*P* = 0.01; Figs. 5g and h).

Inversely, overexpression of *HOXB-AS3* in K-562 cells led to an increase in the abundance of the transcribed rRNA compared with the empty vector control (*P* < 0.001; Fig. 5i). Overexpression of *HOXB-AS3* in K-562 cells also led to increased occupancy of the rDNA promoter by POLR1A (*P* = 0.002; Fig. 5j). Further, we conducted experiments with a luciferase-reporter system of POLR1A activity described previously^43^, which consists of an empty control (pIRES-Luc) and the rDNA promoter-containing (pHrD-IRES-Luc) reporter vectors. K-562 cells transfected with the pHrD-IRES-Luc showed an approximately 10-fold increase in the relative reporter activity, compared with cells transfected with the pIRES-Luc. This increase was significantly higher when K-562 cells were concomitantly transfected with a *HOXB-AS3*-overexpressing vector versus an empty pcDNA3 vector control (*P* = 0.002; Fig. 5k). Finally, *HOXB-AS3* overexpression led to an increase in the de novo protein synthesis of the transfected K-562 cells (*P* = 0.001; Fig. 5l).

### *HOXB-AS3* guides EBP1 to the rDNA locus

We found that *HOXB-AS3* expression levels impacted on the interaction of EBP1 with NPM1 and affected the EBP1-NPM1 complex formation. As EBP1 was shown to directly bind to *HOXB-AS3*, while NPM1 was not (either in the RAP screening or in subsequent RIP experiments), we hypothesized that *HOXB-AS3* could have a guide-RNA function that regulates the localization of EBP1 to the rDNA locus.

To test this hypothesis, we studied the interaction of EBP1 with the rDNA promoter in the presence or absence of *HOXB-AS3*. *HOXB-AS3* KD reduced the amount of EBP1 that was bound to the rDNA promoter, as measured by ChIP assays (*P* = 0.02; Fig. 5m). This effect was rDNA specific and binding of EBP1 on the *E2F1* gene promoter, which has previously identified as an EBP1 target^44^ was not affected by *HOXB-AS3* KD (Supplementary Fig. 11). We also evaluated the occupancy of the rDNA promoter by the NPM1 protein and found that *HOXB-AS3* KD led to reduction in the amount of NPM1 that interacted with the rDNA chromatin (Supplementary Fig. 12). In addition, RAP-DNA experiments showed a significant enrichment of the rDNA promoter region in the *HOXB-AS3*-bound DNA compared with control samples (*P* = 0.02; Fig. 5n), while no difference in enrichment of the *ACTB* promoter (which served as a negative control) was noted.

### Characterization of the *HOXB-AS3*-EBP1 interaction

**Fig. 6:**
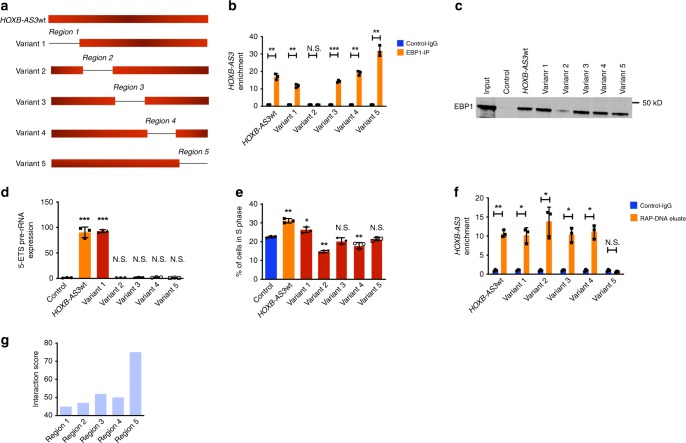
Characterization of the *HOXB-AS3* region which mediates the interaction with EBP1. **a** Schematic representation of truncated *HOXB-AS3* variants 1–5, compared with wild-type *HOXB-AS3* (*HOXB-AS3*wt). The truncated regions are also annotated (1–5). **b** EBP1-targeting RIP experiments in K-562 cells overexpressing *HOXB-AS3*wt or truncated *HOXB-AS3* variants 1–5. The amount of *HOXB-AS3* that interacted with EBP1 is depicted as enrichment in comparison to the rabbit IgG control. *P < 0.05, **P < 0.005, ***P < 0.001, N.S., not significant. **c** Western blotting for the EBP1 protein after incubation of nuclear lysates of K-562 cells with biotinylated *HOXB-AS3*wt or truncated *HOXB-AS3* variants 1–5. **d** Relative pre-rRNA expression in K-562 cells overexpressing *HOXB-AS3*wt or truncated *HOXB-AS3* variants 1–5. ***P < 0.005, ****P < 0.001, N.S., not significant. **e** Cell-cycle analysis in K-562 cells transfected with empty vector, *HOXB-AS3*wt*-* or truncated *HOXB-AS3-*overexpressing vectors. Percentages of cells in the S Phase are shown. **P < 0.01, ****P < 0.001, N.S., not significant. N.S., not significant. **f** Enrichment of 28S rDNA promoter region in RAP-DNA-based isolation of chromatin in K-562 cells transfected with *HOXB-AS3*wt*-* or truncated *HOXB-AS3-*overexpressing vectors. Results are depicted as fold change of enrichment in samples treated with control versus *HOXB-AS3*-capturing biotinylated probes. *P < 0.05, **P < 0.01, N.S., not significant. **g** In silico analysis of the interaction between the *HOXB-AS3* regions and the rDNA promoter. *P* values were calculated using paired two-sided *t*-tests. In the figures, heights of boxplots indicate mean values with standard deviation. Error bars indicate highest and lowest values in each population. Source data are provided as a Source Data file.

To identify specific regions of *HOXB-AS3* that mediate the *HOXB-AS3*-EBP1 interaction, we generated five truncated variants, each of which sequentially lacked ~100 nucleotides (i.e., regions 1–5), compared with the wild-type *HOXB-AS3* sequence (*HOXB-AS3*wt; Fig. 6a). We overexpressed the *HOXB-AS3*wt and the *HOXB-AS3* truncated variants in K-562 cells and then conducted RIP experiments for the EBP1 protein. All truncated variants were expressed at similar levels (Supplementary Fig. 13). In these experiments variant 2, which lacks region 2 (nucleotides 95–195 of *HOXB-AS3*wt), showed decreased capacity to interact with EBP1 (Fig. 6b). The function of the region 2 as a protein binding site was specific for EBP1; other *HOXB-AS3* binding proteins that were tested (NONO, PHB1) showed different patterns of interaction with the *HOXB-AS3* truncated variants (Supplementary Fig. 14).

Moreover, we used the T7 RNA polymerase to generate biotinylated transcripts of the *HOXB-AS3*wt or the *HOXB-AS3* truncated variants. After incubating biotinylated transcripts with nuclear lysates of K-562 cells, we examined their association with the EBP1 protein. In agreement with the RIP assays, the variant 2 bound to a lesser amount of the EBP1 protein, compared with the *HOXB-AS3*wt and the other *HOXB-AS3* truncated variants (Fig. 6c).

Overexpression experiments of the *HOXB-AS3*wt and truncated variants in K-562 cells showed that only the full *HOXB-AS3* sequence and variant 1 increased the abundance of rRNA and generated a proliferative phenotype (Figs. 6d and e).

To further study the mechanistic basis of the interaction *HOXB-AS3* with the rDNA promoter we performed RAP-DNA experiments in K-562 cells that were transfected with either the *HOXB-AS3*wt or each of the truncated *HOXB-AS3* variants. We captured the *HOXB-AS3* transcripts and interrogated the *HOXB-AS3*-interacting chromatin for enrichment of the rDNA promoter. Deletion of region 5 of *HOXB-AS3* abrogated the enrichment of the rDNA promoter sequence that was observed with *HOXB-AS3*wt and the other truncated variants, indicating that region 5 is necessary for the interaction between *HOXB-AS3* and the rDNA promoter (Fig. 6f). Furthermore, we used an in silico approach [i.e., the European Bioinformatics Institute Sequence Aligner Tool (SAT)] to analyze the interaction between the nucleotide sequences of the different *HOXB-AS3* regions and the rDNA promoter. While all tested regions showed some level of complementarity and interaction, region 5 was predicted to interact most avidly with the rDNA promoter, as indicated by the highest SAT score (Fig. 6g).

## Discussion

Deregulation of lncRNA expression is gaining recognition for its important role in cancer pathogenesis. *NPM1*mut-associated lncRNA signatures in younger and older adults with CN-AML have previously been identified^24,25^, but the functional significance of the identified lncRNA transcripts was unknown. *HOXB-AS3*, a lncRNA embedded in the *HOXB* locus, was among the most highly upregulated lncRNAs in *NPM1*mut AML patients. Here, we provide evidence that the upregulation of *HOXB-AS3* is driven by the presence of *NPM1* mutations and that depletion of *HOXB-AS3* has an anti-leukemic effect.

Knockdown experiments of *HOXB-AS3* in vitro, resulted in a consistent decrease in the proliferating capacity of the leukemic blasts. In contrast, overexpression of *HOXB-AS3* led to an increase in the proliferative fraction of leukemic cells. Knockdown of *HOXB-AS3* in a murine PDX model showed an anti-leukemic effect and led to prolongation of survival of the treated mice. It is noteworthy that the effect of the in vitro KD of *HOXB-AS3* was less pronounced than the potent anti-leukemic effect of in vivo *HOXB-AS3* targeting. Among possible reasons that could account for this difference is the fact that the in vitro experiments evaluated a transient KD of the *HOXB-AS3* transcript, whereas during the in vivo experiments treatment with anti-*HOXB-AS3* gapmers was continuous. Moreover, the interaction with the bone marrow microenvironment, which is present in the in vivo studies but cannot be fully recreated in vitro, could also potentially impact on the effects of *HOXB-AS3* depletion. Nevertheless, the phenotypic effects and the molecular pathways that were affected by the downregulation of *HOXB-AS3* were concordant in both experimental settings.

To identify the mechanism by which *HOXB-AS3* expression affects cell proliferation, we first evaluated the effect of *HOXB-AS3* KD on the expression levels of other *HOX* genes. Surprisingly, *HOXB-AS3* KD did not significantly affect the mRNA levels of any of the *HOX* genes tested or the level of HOXA9 protein. This distinguishes *HOXB-AS3* from the majority of the other *HOX* loci-embedded lncRNAs, which have been shown to act as regulators of *HOX* gene expression. We then focused on the interactions of *HOXB-AS3* with the proteome of OCI-AML3 cells. We performed RNA pull-down experiments followed by tandem mass spectrometry analyses and identified the EBP1 protein as an avid binder of *HOXB-AS3*. EBP1 is an RNA-binding protein, which has been previously shown to regulate the proliferative capacity of cancer cells^40^. EBP1 has also been implicated in rRNA transcription and ribosome biogenesis both independently^42^ and via its interaction with NPM1^41^.

In health and disease, rRNA transcription is a highly dynamic process that is tightly regulated; the multiple copies of the rDNA across the genome allow the cells to fine-tune and adjust the output of rRNA according to external stimuli and the availability of nutrients^45^. Importantly, deregulation of rRNA transcription is a molecular pathway, which is implicated in cell proliferation^46^ and is relevant for leukemogenesis^47^. Transcriptional activity of the rDNA locus and ribosome biogenesis are processes dependent on functional *NPM1* alleles. The C-terminus-mutated NPM1 protein, which is the result of the AML-related mutations in the *NPM1* gene, does not associate with the rDNA chromatin^48^ and is translocated (together with a fraction of bound wild-type NPM1 protein) to the cytoplasm^49^. The reduced amount of residual NPM1 in the nucleolus is thought to affect nucleolar integrity and pose a molecular vulnerability in the *NPM1*mut AML cells^49^. We therefore hypothesized that in the presence of heterozygous *NPM1* mutations, a potential regulatory role of the *HOXB-AS3*-EBP1 complex in rRNA transcription could have important functional significance.

**Fig. 7:**
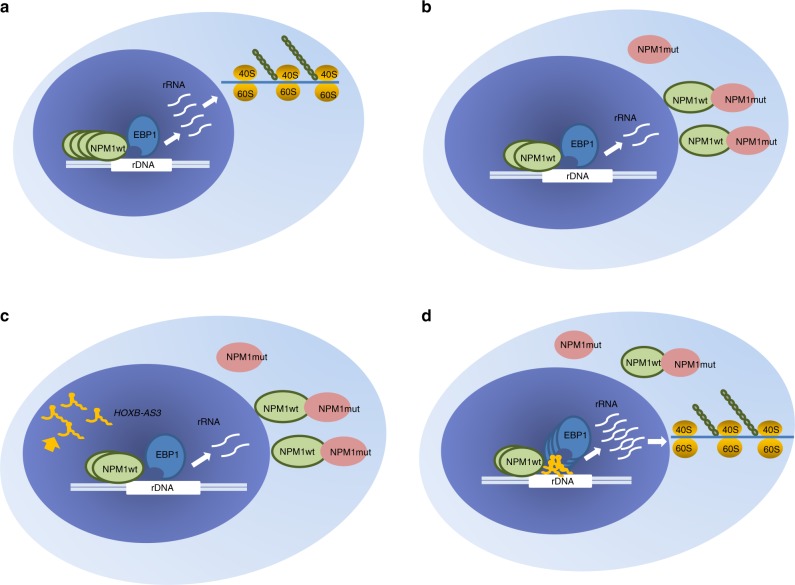
Schematic depiction of the proposed functional role of *HOXB-AS3* in *NPM1*mut AML. **a** Healthy state. NPM1 protein is in its wild-type configuration, resides in the nucleolus and regulates ribosome biogenesis. **b** *NPM1* mutations lead to a reduction of the amount of NPM1 protein in the nucleolus and a reduction in the total amount of the transcribed rRNA. **c** *NPM1* mutations also lead to aberrant upregulation of the *HOXB-AS3* lncRNA. **d** *HOXB-AS3* interacts with and guides EBP1 to the nucleolus. The increased amount of EBP1 interacts with the residual NPM1 and increases the output of transcribed rRNA.

Indeed, we could show that in *NPM1*mut AML blasts, *HOXB-AS3* affects the interaction of EBP1 with NPM1 in the nucleus. Upon depletion of *HOXB-AS3* in OCI-AML3 cells, a lesser amount of NPM1 was found to interact with EBP1. In addition, we found that *HOXB-AS3* KD impacts on the rRNA transcription and, subsequently, the process of protein synthesis in AML blasts. In this sense, the functional role of *HOXB-AS3*, in the context of heterozygous *NPM1* mutations, could be regarded as a compensatory mechanism; in parallel to translocating NPM1 to the cytoplasm, the presence of *NPM1* mutations causes the aberrant overexpression of the *HOXB-AS3* lncRNA. *HOXB-AS3* interacts with and guides EBP1 to the rDNA locus and augments the interaction of EBP1 with the residual NPM1 in the nucleolus. In this manner, *HOXB-AS3* helps maintain adequate amounts of rRNA and maximize the efficiency of the protein translating machinery in the metabolically demanding state of constant proliferation (Fig. 7a–d).

Huang et al.^50^ have recently published an elegant study on the functional significance of *HOXB-AS3* expression in colorectal cancer cells. They identified a small peptide encoded by the NR_033201.2 variant of *HOXB-AS3*, which has a tumor suppressive role. While recent studies highlight the dual nature of RNA transcripts that deploy both production of peptides and non-protein-coding mechanisms to exert their effects on cells^51^, our data do not support that this is the case for *HOXB-AS3* in AML. We found the *HOXB-AS3* lncRNA to be upregulated in *NPM1*mut leukemic blasts and to be absent in healthy BM cells, whereas, in colorectal cancer, it was the downregulation of *HOXB-AS3* that associated with aggressive malignant phenotype. Moreover, we show that the *HOXB-AS3* lncRNA is primarily located in the nucleus of AML blasts and found no evidence that it associates with polysomal fractions. Our RAP experiments also showed no enrichment of ribosomal proteins in the *HOXB-AS3*-eluates compared with the *U1*-pull-downs. In addition, it is worth pointing out that the *HOXB-AS3* transcript variant that we found to be functionally relevant in AML shows limited overlap with the *HOXB-AS3* variant that was characterized in colorectal cancer (Supplementary Fig. 1). Collectively, these data suggest that in *NPM1*mut AML blasts *HOXB-AS3* acts as a genuinely non-protein-coding RNA and displays a different mechanism of function from the one that has been described in colorectal cancer.

The in vivo *HOXB-AS3* KD experiments that we conducted to evaluate the functional relevance of *HOXB-AS3* in *NPM1*mut AML also underscore the potential therapeutic value of *HOXB-AS3* depletion. The absence of *HOXB-AS3* expression in healthy hematopoietic BM cells makes it an attractive therapeutic target. We optimized a LNP-based method for packaging and delivering anti-*HOXB-AS3* gapmers in vivo with no significant toxicities. Depletion of *HOXB-AS3* led to significant prolongation of the overall survival of mice transplanted with AML patient blasts. To our knowledge, this is one of the few preclinical models, in which in vivo targeting of a lncRNA transcript as single-agent therapy yielded therapeutic results^52^. Despite the preliminary nature of this data and the additional experiments that are warranted (to define optimal dosing, treatment schedule, delivery method etc.), we believe that the in vivo anti-leukemic effect of *HOXB-AS3* depletion is promising and supports the feasibility of lncRNA targeting in the treatment of AML.

## Methods

### Acute myeloid leukemia patients and healthy BM donors

For association analyses of *HOXB-AS3* expression by *NPM1* mutational status, publicly available data of CN-AML patients were further analyzed. For in vitro and in vivo functional experiments, leukapheresis samples of AML patients were used. The cytogenetic and gene mutational profiles of all patients, whose samples were used in functional experiments, are provided in Supplementary Table 2. BM-isolated mononuclear cells from six healthy donors were purchased from an institutional Biobank Core Facility. For in vitro and in vivo functional experiments, leukapheresis samples of AML patients deposited in the Leukemia Tissue Bank of The Ohio State University were used. All patients and healthy donors were de-identified and had provided written informed consent for the use of their biologic specimens for research purposes according to the Declaration of Helsinki. All relevant ethical regulations for work with human participants were in accordance with the Institutional Review Board (IRB), protocol number 1997C0194 of The Ohio State University.

### Acute myeloid leukemia cell lines

Kasumi-1, KG-1a, K-562, MV-4-11, and THP-1 cells were purchased from the American Type Culture Collection (ATCC) cell repository. MOLM-13 and OCI-AML3 cells were purchased from the Leibniz Institute DSMZ-German Collection of Microorganisms and Cell Cultures. The HEK 293T/17 cells were purchased for the ATCC cell repository. Presence of a type A *NPM1* mutations in OCI-AML3 cells was confirmed by PCR amplification and Sanger sequencing. All other cell lines were recently obtained from the respective cell repositories and were not further characterized.

### Culture conditions of acute myeloid leukemia patient blasts and cell lines

For in vitro experiments AML patient blasts were cultured in StemSpan SFEM medium, supplemented with 10% BIT serum substitute (STEMCELL Technologies Inc.), 1% antibiotic-antimycotic solution (Anti-anti, Gibco), 1% L-Glutamine (Gibco) and cytokines [rmScf (100 ng/ml), rhTPO (50 ng/ml), rhFLT3-ligand (40 ng/ml), rhIL-3 (10 ng/ml), and rhIL-6 (10 ng/ml)]. All cytokines were purchased from PeproTech. All cultures were performed in 37 °C/5% CO_2_.

KG-1a, K-562, MV-4-11, MOLM-13, OCI-AML3, and THP-1 cells were cultured in Roswell Park Memorial Institute (RPMI-1640) medium (Sigma), supplemented with 10% fetal bovine serum (FBS, Sigma), 1% antibiotic-antimycotic, and 1% L-glutamine. Kasumi-1 cells were cultured in similar medium substituted with 20% FBS. HEK 293T/17 cells were grown in Dulbecco’s modified Eagle medium (DMEM) medium (Sigma), supplemented with 10% FBS and 1% antibiotic-antimycotic. All cell line cultures were performed in 37 °C/5% CO_2_.

### Human AML patient-derived xenografts

For in vivo studies, NOD.Cg-*Prkdc*^*scid*^ *Il2rgtm*^1W*jl*^/SzJ (NSG) mice were used as recipients of *NPM1*mut AML patient blasts. Mice were pretreated with busulfan [20 mg/kg weight with intraperitoneal (i.p.) injections] and blasts of each patient were injected via tail vein 1-day post treatment. Female mice of 6–8 weeks of age were used for experiments. All animal studies were conducted according to protocols approved by the Institutional Animal Care and Use Committees of The Ohio State University (IRB protocol #2018C0072).

On day 10 post transplantation, NSG mice were assigned to groups to receive anti-*HOXB-AS3* or non-targeting-control treatment. Assignment to two groups was made based on the weight of each mouse prior to gapmer treatment and caution was taken so that weight distributions were similar between the groups.

Blood samples were periodically acquired and analyzed by flow cytometry [stained with antibodies against human CD45 and mouse 45.1 (BD Biosciences), at 1:1000 dilution]. Only mice that showed ≥5% of human leukemic blasts were included in the study.

### RNA isolation and real-time quantitative PCR

RNA isolation was performed with the Trizol reagent according to the instructions of the manufacturer (Invitrogen). Reverse transcription was performed with the Superscript III single strand RT kit, according to the instructions of the manufacturer (Invitrogen). All custom-designed real-time qPCR (RT-qPCR) primers were synthesized by IDT Inc. as a for-fee service. RT-qPCR experiments were conducted in a RT-qPCR machine (Applied Biosystems, 7500 Real Time PCR System) in the conditions specified by the manufacturer.

### Flow cytometry analyses

Cell-cycle and apoptosis analyses were performed with the BrdU and the Annexin/PI staining kits of BD Pharmingen. Experiments were analyzed with flow cytometry on an LSRII instrument, according to the instructions of the manufacturer. Gating strategies are exemplified in Supplementary Fig. 15.

### Cell fractionation, polysome profiling, and isolation

For the separation of OCI-AML3 cells in nuclear and cytoplasmic fractions 3 × 10^6^ cells were harvested and lysed by use of the NePER cell fractionation kit (Thermo Scientific) according to the instructions of the manufacturer.

For the isolation of polysome fractions and polysome profiling, 10×10^6^ OCI-AML3 cells were grown at a concentration of 5 × 10^5^ cells/ml. Prior to lysis, a freshly prepared DMSO-solution of cycloheximide was added to the medium to a final concentration of 100 µg/ml. Cells were incubated for 10 min at 37 °C/5% CO_2_, washed in cycloheximide-containing PBS (100 µg/ml) and lysed in lysis buffer [(50 mM Tris-HCl pH 7.5, 10 mM KCl; 10 mM MgCl_2_, 150 mM NaCl, 2 mM DTT, 0.5 mM PMSF, 200 μg/l cycloheximide, 0.2% IGEPAL, and protease inhibitors: leupeptin (Sigma-Aldrich), pepstatin A (Sigma-Aldrich), and aprotinin (Sigma-Aldrich)]. Lysates were incubated on ice for 10 min and cleared by centrifugation in a tabletop microcentrifuge (16,000 × *g*, 10 min, 4 °C). The supernatants were collected into fresh tubes and flash frozen until polysome isolation or profiling was conducted.

Cytoplasmic extracts were thawed on ice. The equivalent of 4.5 million OCI-AML33 cell lysates was layered onto each 11 ml of 10–50% linear sucrose gradient. Gradients were then centrifuged (35,000 rpm, 3 h, 4 °C) in a Sorvall-TH641 rotor at 4 °C. Gradient fractions were collected at 500 µl intervals with continuous measurement of UV absorbance at 254 nm.

For measurements of RNA transcripts, each isolated polysomal fraction was mixed with 1 ml of Trizol reagent and standard RNA extractions were performed. RNA samples were pooled together prior to reverse transcription and RT-qPCR. Cell pellets of 0.45 million cells were lysed with Trizol reagent and standard RNA. The unfractionated cells were used as input material, to determine the relative abundance of the transcripts on each isolated fraction of the sucrose gradient.

### Gene knockdowns and overexpression

For the knockdown (KD) of genes in OCI-AML3 cells and samples from *NPM1*mut AML patients we used custom-designed locked nuclei acid-modified RNaseH recruiting oligonucleotides (gapmers, Exicon A/S). The sequences of the gapmers are provided in Supplementary Table 1. For the in vitro delivery of the gapmers, the Nucleofector device (Lonza) and the corresponding reagents (Solution T for OCI-AML3 cells and human Monocyte Solution for AML blasts, Lonza) were used according to the instructions of the manufacturer (program X-001 for OCI-AML3 cells and program Y-001 for patient blasts). Anti-*HOXB-AS3*, anti-*NPM1*mut or non-targeting control gapmers were delivered at a final concentration of 500 nM.

For the in vivo KD of the *HOXB-AS3* lncRNA, *HOXB-AS3*-targeting gapmers or non-targeting controls were packaged into cationic lipid nanoparticles (LNPs) conjugated with human transferrin. In brief, the LNPs consisted of 1, 2-dioleoyl-3-trimethylammonium-propane (DOTAP) and 1,2-dioleoyl-sn-glycero-3-phosphocholine (DOPC) at a molar ratio of 1-to-1. DOTAP (18.8 mg) and DOPC (21.2 mg) were dissolved in 1 ml of ethanol and were then injected into 9 ml of HEPES buffer (20 mM, pH 7.4). After 5 min of incubation in a water bath sonicator, the nanoparticles were passed through a 0.22 µm sterile filter. Solutions of gapmers were mixed with lipids at a 1-to-10 weight ratio, vortexed and incubated at room temperature for 10 min. The lipid-oligo formulation was then mixed with a human transferrin solution. The mixtures were vortexed and sonicated in a water bath sonicator for 10 min and incubated for an hour at 37 °C. For the first week of treatment mice were treated with i.v. injections on days 1, 3, and 5 (of 5 mg of gapmers/kg weight) and i.p. injections on days 2 and 4 (of 10 mg of gapmers/kg weight). The mice received only i.p. injections (5/week) for the remainder of the treatment course.

Delivery of the control or *HOXB-AS3*-overexpressing pcDNA3 vectors to K-562 cells was performed with electroporation with the Nucleofector device according to the instructions of the manufacturer (Solution V, program T-016). Two micrograms of vector were used per reaction.

### In vitro colony-forming assays

For colony formation unit assays with AML cell lines, the cells were electroporated, cultured for 24 h and then mixed with basic pre-warmed methylcellulose-based medium without cytokines (MethoCult H4100, STEMCELL Technologies Inc), supplemented with 10% FBS. AML cells were seeded at a concentration of 1000 cells/ml, and colonies were counted on day 14.

For CFU assays with human AML patient blasts, the cells were electroporated, cultured for 24 h (as described in the section detailing culture conditions) and then mixed with pre-warmed methylcellulose-based medium (Methocult H4034 Optimum, STEMCELL Technologies Inc.) according to the instructions of the manufacturer. Leukemic blasts were seeded at a concentration of 50,000 blasts/ml of medium and colonies were counted on day 14.

### CRISPR/Cas9 silencing of *Hoxb5os* in *Npm1*^cA/+^ cell lines

For disruption of the *Hoxb5os* transcript with the CRISPR/CAs9 technique, guide RNAs targeting two separate regions of the transcript were cloned into the dual guide expressing pKLV2-.2-h7SHgRNA5(SapI)-U6gRNA5(BbsI)-PGKpuroBFP-W lentiviral vectors as previously described^33^. BM-derived AML cells from Rosa26-EF1-Cas9 mice were cultured in vitro in the presence of cytokines. Disruption of individual candidate genes was performed by transduction with lentivirus expressing gene-specific guide RNAs (gRNA) and blue fluorescent protein (BFP). The impact of gene disruption on AML cell growth was determined using competitive co-culture of transduced (BFP+) versus non-transduced (BFP−) cells. In brief, transduced cells were allowed to grow in the presence of non-transduced cells while the percentage of BFP+ cells was measured by flow cytometry on days 4, 8, 11, and 15. For the depiction of the results, the percentage of BFP+ cells on day 4 was used as reference. Based on previously published results^34^, gRNAs targeting the *Aurkb* gene were used as a pan-essential positive control. Sequences of the gRNAs used are provided in Supplementary Table 1d.

### Transcriptome analyses

Libraries for RNA-seq experiments were generated as follows: after Trizol-based RNA extraction, total RNA extracted from blasts of four different *NPM1*mut AML patients and OCI-AML3 cells was used for library generation. Patient AML blasts were treated with anti-*HOXB-AS3* or non-targeting scramble control gapmers and harvested at 24 h post electroporation. OCI-AML3 cells were treated similarly and harvested at 12, 24, and 48 h post electroporation. Samples were assessed for quality on an Agilent 2100 Bioanalyzer (BioA) using the RNA 6000 Nanochip and for quantity on a Qubit 2.0 Fluorometer (Agilent Technologies) using the RNA HS Assay Kit. Samples with an RNA integrity number (RIN) >7, with no visible sign of genomic DNA (gDNA) contamination and a concentration of >40 ng/μl were used for mRNA library generation. RNA-seq libraries were prepared using the Illumina TruSeq Stranded mRNA Sample Prep Kit with poly-A-tail selection of the RNA transcripts (#RS1222201) according to the instructions of the manufacturer. Sequencing was performed with the Illumina HiSeq 2500 system using the HiSeq version 3 sequencing reagents to an approximate cluster density of 800,000/mm^2^. Image analysis, base calling, error estimation, and quality thresholds were performed using the HiSeq Controller Software (version 2.2.38) and the Real Time Analyzer (RTA) software (version 1.18.64).

Analysis of RNA sequencing data were performed as follows: transcript abundance was quantified from the RNA-seq data using Kallisto^53^, with a reference transcriptome consisting of Homo sapiens GRCh38 protein-encoding and non-coding transcripts except rRNAs; the strand-specific option of “first read reverse” was chosen; 100 bootstrap subsamples were generated for each RNA-Seq sample to estimate inferential variance. Gene-level differential expression analysis was performed on the kallisto output using linear models via Sleuth^54^ (10.1038/nmeth.4324). For patient data, patient identifier was included as a fixed effect to conduct a paired analysis; for cell line data, repeated measures ANOVA was used to test for differences between scramble and *HOXB-AS3* KD. Likelihood ratio tests were performed to determine whether each gene showed significant differential expression between control (scramble) and *HOXB-AS3* KD treatments. A false discovery rate threshold FDR < 0.05^55^ was used to detect significance in differential expression between scramble and *HOXB-AS3* KD samples.

### RNA-antisense purification experiments

RNA-protein complex pull-down experiments were conducted according to a modified version of the RAP-MS protocol published by McHugh et al.^36^. In brief, AML blasts were cross-linked in batches of 20 × 10^6^ cells via exposure to 8000 J/m^2^ of ultraviolet (UV) irradiation. Cells were lysed in lysis buffer (10 mM Tris-HCl, 500 mM LiCl, 0.5% dodecyl maltoside (DDM), 0.2% sodium dodecyl sulfate (SDS), 0.1% sodium deoxycholate), sonicated and treated with DNAse I (Norgen Biotek). Lysates were mixed with hybridization buffer (10 mM Tris-HCl, 5 mM EDTA, 500 mM LiCl, 0.5% DDM, 0.2% SDS, 0.1% sodium deoxycholate, 4 M urea, 2.5 mM TCEP) and spun down in a tabletop centrifuge (16,000 × *g*, 10 min, 4 °C). The supernatants were pre-cleaned by incubation (intermittent shaking at 1100 rpm, 30 min, 37 °C) with streptavidin-coated magnetic beads (Dynabeads MyOne, Streptavidin C1, Life Technologies). After removal of beads, the lysates were incubated with biotinylated probes (2 μg of probes per 20 × 10^6^ cells) that were complementary to either the *HOXB-AS3* or the *U1* transcripts. The sequences of the biotinylated probes are provided in Table 1. Probes and lysates were allowed to hybridize by incubation for 2 h at 67 °C with intermittent shaking at 1100 rpm. Streptavidin-coated magnetic beads were used for the purification of complexes. The purified complexes were either treated with Proteinase K (New England Biolabs) to digest protein residues or with benzonase nuclease (EMD Millipore) to digest probes and captured RNA and release RNA-bound protein residues from the streptavidin beads. Proteinase K-treated eluates were mixed with elution buffer N-lauroylsarcosine-rich (NLS, Sigma-Aldrich) buffer, heated for 2 min at 95 °C and released from the magnetic beads. Eluted nucleic acids were captured by magnetic SILANE beads (Invitrogen, Dynabeads MyOne), treated with DNase I and resuspended in RNase-free water. The isolated RNA was reversed transcribed into cDNA and analyzed with RT-qPCR, as described above.

Polypeptides in the benzonase endonuclease-treated samples were precipitated by overnight incubation with trichloroacetic acid (Ricca Chemical Company) and centrifugation (16,000 × *g*, 10 min, 4 °C). Polypeptides were then digested with Trypsin (Promega) and Endopeptidase Lys-C (Wako) according to the instructions of the manufacturers, purified with HiPPR detergent removal columns (Life Technologies) and analyzed with liquid chromatography tandem mass spectrometry (LC/MSMS) on a Thermo Fusion Orbitrap Mass Spectrometer using 1 h LC gradient. Peptides isolated from a total of at least 400 × 10^6^ cells were used for each experiment. Mass spectrometry analyses were performed in triplicates and the results were filtered for number and quality of identified peptides. Proteins with two or more individual peptides identified in at least two of the analyzed samples, and a quality score of ≥30 were considered as putative interactors. The identified potential interactors were further filtered on the basis of previously reported RNA-binding capacity^56,57^ and were analyzed comparatively to identify putative *HOXB-AS3*- and *U1*-specific protein interactors.

For the RAP-DNA experiments, the same experimental principles were used for capturing the targeted transcripts with some modifications. Cross-linking was performed with a combination of formaldehyde and DSG treatment, as previously described^58^. Biotinylated probes with sequences identical to that of *HOXB-AS3* (which were expected to hybridize to the complementary DNA strain of the *HOXB-AS3* gene, but not the *HOXB-AS3* transcript) were used as controls. Probes were hybridized with lysates for 3 h, at 37 °C with intermittent shaking (1100 rpm).

### RNA immunoprecipitation and protein co-immunoprecipitation experiments

RIP experiments and co-immunoprecipitation (co-IP) experiments were conducted in nuclear lysates according to protocols provided by Abcam (http://www.abcam.com/epigenetics/rna-immunoprecipitation-rip-protocol). For the RIP experiments aimed at the validation of the RAP screening, either OCI-AML3 cell lysates and the corresponding antibodies [i.e., anti-EBP1 (Abcam), anti-NONO (Abcam), anti-PHB1 (Cell Signalling Technologies), anti-DHX9 (Abcam), anti-MATR3 and anti-SRSF2 (Abcam)] or 293T cells transfected with a *HOXB-AS3* overexpressing vector and a pcDNA3 vector expressing a FLAG-tagged protein [i.e., PARK7, PCBP1 or HNRNPC, in which case an anti-FLAG antibody (Sigma-Aldrich) was used] were analyzed. In both cases, 10 × 10^6^ cells were cross-linked by exposure to UV irradiation (8000 J/m^2^) prior to cell lysis. Cells were then resuspended in 10 ml of lysis Buffer consisting of 6 ml of water, 2 ml of PBS, and 2 ml of Nuclear Isolation Buffer (1.28 M sucrose, 40 mM Tris-HCl pH 7.5, 20 mM MgCl_2_, 4% Triton X-100). Nuclei were pelleted by centrifugation (2500 × *g*, 15 min, 4 °C), and were then resuspended in 1 ml of RIP Buffer (150 mM KCl, 25 mM Tris pH 7.4, 5 mM EDTA, 0.5 mM DTT, 0.5% NP40 plus RNase and Protease inhibitors). Cell pellets were sonicated with a microtip sonicator (pulse-sonication at 5 W for 10 s followed by a 20-s interval, repeated for a total of three times) and spun down for 10 min at 16,000 × *g* at 4 °C. Supernatants were mixed with magnetic A/G beads (Pierce Biotechnology), pre-coated with antibodies and incubated overnight at 4 °C, rotating. For the co-IP experiments antibodies were cross-linked to beads by use of dimethyl pimelimidate (DMP) according to the instructions provided by Abcam (http://www.abcam.com/protocols/cross-linking-antibodies-to-beads-protocol). After incubation, beads were magnetically separated, washed and mixed with either proteinase K-containing solution (New England Biolabs) for RIP experiments, or with equal volumes of beta-mercaptoethanol-containing Lamelli buffer for co-IP experiments.

After proteinase K-treatment (incubation for 1 h at 52 °C) samples were mixed with 1 ml of Trizol reagent. Standard Trizol-based RNA isolation was subsequently performed. For co-IP experiments, the beads were denatured at 99 °C for 10 min. Following magnetic removal of beads, the eluates were directly loaded onto SDS-PAGE gels and separated by electrophoresis.

### Chromatin-immunoprecipitation experiments

For chromatin-immunoprecipitation experiments, antibodies against RNA Polymerase I (POLR1A, Abcam) and against EBP1 (Abcam) were used. To isolate nucleolar chromatin we used the experimental approach described by O’Sullivan et al.^59^. In brief, cells were cross-linked with formaldehyde (incubation with 0.25% solution for 10 min) and washed with PBS. After centrifugation at 200 × *g* for 5 min, cell pellets were resuspended in 1 ml of high-magnesium buffer (10 mM HEPES Buffer, 0.88 M sucrose, 12 mM MgCl_2_, and 1 mM DTT plus protease inhibitors). Nucleoli were released by sonication on ice (three bursts of 10 s each at 20 W). Nucleoli were pelleted by centrifugation in a tabletop microcentrifuge (15,000 × *g*, 10 min, 4 °C), and the pellets were resuspended in 1 ml of low-magnesium buffer (10 mM HEPES, 0.88 M sucrose, 1 mM MgCl_2_, and 1 mM DTT plus protease inhibitors). Nucleoli were subject to further sonication on ice (10 s at full power) and pelleted as before. Isolated nucleoli were resuspended in 0.2 ml of 20/2TE (20 mM Tris, 2 mM EDTA) plus 1/10 volume of 20% SDS. Following incubation at 37 °C for 15 min, 800 µl of 20/2TE were added, and the solutions were sonicated (three to four bursts of 5 s each at full power). The resulting sheared nucleolar chromatin was centrifuged in a microcentrifuge (15,000 × *g*, 10 min, 4 °C), and the nucleolar chromatin supernatant was used immediately in ChIP assays.

POLR1A or EBP1 antibodies were incubated with magnetic agarose A/G beads for 1 h at room temperature and washed twice with PBS. Agarose beads were blocked by incubation in 2% BSA and salmon sperm containing solution. Cell lysates were pre-cleaned with blocked agarose beads. Antibody-coated beads were incubated overnight with nucleolar lysates. Chromatin immunoprecipitates were purified with phenol:chloroform:isoamyl alcohol (25:24:1) according to the instructions of the manufacturer (Thermo Scientific) and analyzed with the Simple Chip 28S rDNA assay (Cell Signaling Technology).

### Western blots

For the detection of proteins, cells were lysed with 50–100 µl of RIPA buffer (Pierce Biotechnology), supplemented with proteinase and phosphatase inhibitors (EMD Millipore). Samples were incubated on ice for 30 min and were intermittently vortexed and sonicated (three bursts of 10 s at 3 W). Lysates were cleared by centrifugation in a tabletop centrifuge (16,000 × *g*, 10 min, 4 °C). Supernatants were transferred to fresh tubes and protein concentration was measured by means of Bradford protein assays.

Equal amounts of proteins were mixed with Lamelli Buffer (Bio-Rad) supplemented with 5% beta-mercaptoethanol (Sigma-Aldrich). Samples were denatured by incubation at 99 °C for 10 min. Samples were then loaded on sodium dodecyl sulfate polyacrylamide gel electrophoresis (SDS-PAGE) gels and subsequently weight-separated by electrophoresis. Separated proteins were then transferred to Nitrocellulose membranes with the Fast-Transfer apparatus and kit (Bio-Rad). All antibodies used in this study are listed in Supplementary Table 4. Unless otherwise stated, primary antibodies were used in a 1:1000 and secondary antibodies in a 1:5000 dilution.

### rRNA transcription reporter assays

K-562 cells were concomitantly transfected with (i) the pRL-TK Renilla Luciferase (0.1 μg), (ii) the pGL3-IRES-Luc or the pHrD-IRES-Luc (0.9 μg) and (iii) the empty pcDNA3 or the pcDNA3-*HOXB-AS3* overexpressing vectors (1.5 μg). Transfected cells were incubated for 72 h in 37 °C/5% CO_2_ and were then lysed in lysis buffer (Promega), according to the instructions of the manufacturer. Luciferase activity was measured using the Dual-Glo Luciferase Assay kit (Promega) in a luminometer (Glomax Luminometer, Promega).

### De novo protein synthesis assays

To evaluate the effect of *HOXB-AS3* KD in de novo protein synthesis we used an O-proargyl-puromycin (OPP) labeling kit according to the instructions of the manufacturer (Cayman Chemicals). We incubated cultured cells in OPP containing medium (2 h for OCI-AML3 cells and patient blasts, and 45 min for K-562 cells), in order to label newly synthetized peptides. Cells were then fixed with 10% formalin, washed and conjugated with an Alexa-Fluor 488 fluorochrome. Cycloheximide-treated samples were used as negative controls. Samples were analyzed immediately after preparation by flow cytometry analyses.

### Generation of truncated *HOXB-AS3* variants

To generate truncated *HOXB-AS3* variants, which sequentially lacked ~100 nucleotides compared with the wild-type transcript, we designed primers (Supplementary Table 1f) and conducted overlap PCR experiments using Phusion Polymerase according to the instructions of the manufacturer (New England Biolabs). Amplicons were cloned into TOPO-blunt end vectors (Invitrogen) and sequenced. Vectors containing validated amplicons were digested with HindIII and BamHI and cloned into pcDNA3 vectors as described above.

### Biotinylation of wild-type *HOXB-AS3* and the truncated *HOXB-AS3* variants

To generate biotinylated *HOXB-AS3*wt or mutated *HOXB-AS3* variants, the pcDNA3 vectors containing the respective sequences were digested with the ApaI restriction enzyme (New England Biolabs). Digested vectors were weight-separated by agarose gel electrophoresis, visualized and purified from the gel with the Qiagen gel extraction kit. Purified vectors were used as template in an overnight T7 RNA transcriptase-mediated reaction (New England Biolabs), which was conducted in the presence of biotinylated ribonucleotides (Roche), according to the instructions of the manufacturer.

Generated biotinylated RNA was purified with a Trizol extraction and measured on a Nanodrop device. Equal amounts of biotinylated RNA were mixed with 100 µl of magnetic streptavidin beads (MyOne Silane). Bead-bound biotinylated RNA was mixed and allowed to hybridize with nuclear lysate of K-562 cells (lysates of 10 × 10^6^ cells were used per reaction and were incubated for 30 min at 37 °C). Streptavidin beads were magnetically separated, washed and mixed with beta-mercaptoethanol-containing buffer. The eluates were denatured by incubation at 99 °C for 10 min and were then loaded on SDS-PAGE gels.

### Mass spectrometry analysis

Samples were digested with trypsin and the peptides were analyzed using LC/MSMS on a Thermo Scientific orbitrap Fusion mass spectrometer equipped with an EASY-Spray™ Sources operated in positive ion mode. Samples were separated on an easy spray nano column (PepmapTM RSLC, C18 3 µ 100 A, 75 µm × 150 mm) using a 2D RSLC HPLC system from Thermo Scientific. Mobile phase A was 0.1% formic acid in water and acetonitrile (with 0.1% formic acid) was used as mobile phase B. Flow rate was set at 300 nL/min. MS/MS data were acquired with a spray voltage of 1.7 KV and a capillary temperature of 275 °C is used. The scan sequence of the mass spectrometer was based on the preview mode data dependent TopSpeed™ method. To achieve high mass accuracy MS determination, the full scan was performed at FT mode and the resolution was set at 120,000. EASY-IC was used for internal mass calibration. The AGC Target ion number for FT full scan was set at 4 × 10^5^ ions, maximum ion injection time was set at 50 ms and micro scan number was set at 1. MSn was performed using ion trap mode to ensure the highest signal intensity of MSn spectra using both HCD methods (30%). The AGC Target ion number for ion trap MSn scan was set at 10,000 ions, maximum ion injection time was set at 30 ms and micro scan number was set at 1. Dynamic exclusion is enabled with a repeat count of 1 within 60 s and a low mass width and high mass width of 10 ppm. Sequence information from the MS/MS data were processed by converting the .raw files into a merged file (.mgf) using MS convert (ProteoWizard). The resulting mgf files were searched using Mascot Daemon by Matrix Science version 2.3.2 (Boston, MA) and the database searched against Uniprot Human database (version 12032015). The mass accuracy of the precursor ions were set to 10 ppm, accidental pick of one 13C peaks was also included into the search. The fragment mass tolerance was set to 0.5 Da. Considered variable modifications were oxidation (Met), deamidation (N and Q), and carbamidomethylation (Cys). Four missed cleavages for the enzyme were permitted. A decoy database was also searched to determine the false discovery rate (FDR) and peptides were filtered according to the FDR. The significance threshold was set at *p* < 0.05 and bold red peptides is required for valid peptide identification. Proteins with <1% FDR as well as a minimal of two significant peptides detected were considered as valid proteins.

### Quantification of nucleic acid abundancies

Quantification of RNA transcripts measured with RT-qPCR was performed comparatively, based on the differential threshold cycle (Ct) value method. Ct values of measured transcripts were normalized against Ct values of human *GAPDH* mRNA, human *ACTB* mRNA or murine *Gapdh* Ct values, which were used as reference. To determine the RNA abundance of transcripts in subcellular compartments (i.e., cytoplasmic versus nuclear compartment or polysomal fractions), the Ct values of the measured transcripts were normalized against the respective Ct values detected in the input sample and presented as percent of the amount of transcript in the input sample. For RNA-antisense purification (RAP), RNA-immunoprecipitation and chromatin-immunoprecipitation (ChIP) experiments, abundance of measured RNA or DNA was analyzed either as percent of the input sample or as fold-enrichment of the amplicon in the measured eluate, compared with the respective negative control sample [e.g., for RIP experiments, the normal rabbit IgG antibodies (Millipore Sigma)].

### In silico analysis of *HOXB-AS3* and rDNA promoter interaction

In order to further investigate the interaction of the *HOXB-AS3* lncRNA with the rDNA promoter we used an in silico approach. Specifically, we used the European Bioinformatics Institute Sequence Aligner Tool (SAT), which is an on-line available sequence analyzer (https://www.ebi.ac.uk/Tools/psa/). We examined the strength of interaction between the rDNA promoter and each of the *HOXB-AS3* regions that were deleted in the truncated variants used in the RAP-DNA experiments. The SAT generated a composite score that was based on the level of complementarity between the analyzed sequences (also taking into account the effect of the non-complementary bases). Higher scores were indicative of higher predicted strength of interaction between examined sequences.

### Statistical analyses

For the quantification of nucleic acid abundancies in the RNA-seq data, the Wilcoxon rank sum test was used to compare *HOXB-AS3* expression between *NPM1-*mutated and *NPM1* wild-type patients. For RNA-seq analyses of scramble versus *HOXB-AS3* KD-treated samples, the repeated measures ANOVA test was used to test for differences in gene expression. Likelihood ratio tests were performed to determine whether each gene showed significant differential expression between scramble and *HOXB-AS3* KD treatments. A false discovery rate threshold FDR < 0.05 was used to detect significance in differential expression between scramble and *HOXB-AS3* KD samples.

A *t*-test comparing two independent samples, assuming unequal variances, was used to compare the means of *HOXB-AS3* expression between BM samples from healthy donors, *NPM1*mut and *NPM1*wt AML patients; to compare the means of *Hoxb5os* expression in *Npm1*^WT^ and *Npm1*^cA/+^ mice; and to conduct analyses in AML xenografts that received short-term treatment with scramble or anti-*HOXB-AS3* gapmers. For all other laboratory experiments, a paired *t*-test analysis was used to conduct comparisons. A threshold *P*-value < 0.05 was used to detect significance.

For the long-term in vivo experiment with AML patient xenografts treated with scramble or anti-*HOXB-AS3* gapmers, the estimated probabilities of overall survival were calculated using the Kaplan–Meier method. The log-rank test was used to evaluate differences between survival distributions. All analyses of laboratory experiments were conducted with the GraphPad Prism software.

All data presented in this work (including uncropped and unprocessed scans of the presented blots) are provided in the Source Data file that accompanies the paper.

## Supplementary information

Supplementary Information

## Data Availability

The RNA sequencing datasets that were generated during and/or analyzed during the current study are available in the GEO repository, under the accession number: GSE137851. The proteomics datasets that were generated during and/or analyzed during the current study are available in the ProteomeXchange Consortium via the PRIDE partner repository with the dataset identifier PXD015520. All reagents (e.g., plasmids, oligonucleotides, real-time PCR assays) generated during the current study are available from the corresponding author on reasonable request.

## Source data

Source Data
