# Publisher Correction: The long non-coding RNA *HOXB-AS3* regulates ribosomal RNA transcription in *NPM1*-mutated acute myeloid leukemia

**DOI:** 10.1038/s41467-019-13969-7

**Published:** 2020-01-07

**Authors:** Dimitrios Papaioannou, Andreas Petri, Oliver M. Dovey, Sara Terreri, Eric Wang, Frances A. Collins, Lauren A. Woodward, Allison E. Walker, Deedra Nicolet, Felice Pepe, Prasanthi Kumchala, Marius Bill, Christopher J. Walker, Malith Karunasiri, Krzysztof Mrózek, Miranda L. Gardner, Virginia Camilotto, Nina Zitzer, Jonathan L. Cooper, Xiongwei Cai, Xiaoqing Rong-Mullins, Jessica Kohlschmidt, Kellie J. Archer, Michael A. Freitas, Yi Zheng, Robert J. Lee, Iannis Aifantis, George Vassiliou, Guramrit Singh, Sakari Kauppinen, Clara D. Bloomfield, Adrienne M. Dorrance, Ramiro Garzon

**Affiliations:** 1 The Ohio State University, Comprehensive Cancer Center, Columbus, OH USA; 2 Center for RNA Medicine, Department of Clinical Medicine, Aalborg University, Copenhagen, Denmark; 3 Wellcome Trust Sanger Institute, Wellcome Trust Genome Campus, Cambridge, UK; 4 Institute of Genetics and Biophysics (IGB-ABT), National Council of Research (CNR), Naples, Italy; 5 Department of Pathology, New York University School of Medicine, New York, NY USA; 6 Department of Molecular Genetics, Center for RNA Biology, The Ohio State University, Columbus, OH USA; 7 Alliance for Clinical Trials in Oncology Statistics and Data Center, The Ohio State University, Columbus, OH USA; 8 Department of Cancer Biology and Genetics, The Ohio State University, Columbus, OH USA; 9 Department of Molecular Medicine, University of Pavia, Pavia, Italy; 10 Cancer and Blood Diseases Institute, Cincinnati Children’s Hospital Medical Center, University of Cincinnati, Cincinnati, OH USA; 11 Division of Biostatistics, College of Public Health, The Ohio State University, Columbus, OH USA; 12 Division of Pharmaceutics, College of Pharmacy, The Ohio State University, Columbus, OH USA; 13 Department of Haematology, Cambridge University Hospitals NHS Trust, Cambridge, UK

**Keywords:** Cancer, Molecular biology, Non-coding RNAs, Transcription, Molecular medicine

Correction to: *Nature Communications* 10.1038/s41467-019-13259-2, published online 25 November 2019.

The original version of this Article contained an error in the author affiliations.

Affiliation number 9 incorrectly read “Department of Molecular Medicine, University of Pavia, Pavia, USA”

This has now been corrected in both the PDF and HTML versions of the Article

