# Supplementary material for: The long non-coding RNA HOXB-AS3 regulates ribosomal RNA transcription in NPM1-mutated acute myeloid leukemia
Source: Nat Commun. 2019 Nov 25;10:5351. doi: 10.1038/s41467-019-13259-2 (PMC6877618; doi:10.1038/s41467-019-13259-2)
Supplement: Supplementary file 1 — Supplementary Information [file 41467_2019_13259_MOESM1_ESM.pdf]

SUPPLEMENTARY INFORMATION

The long non-coding RNA *HOXB-AS3* regulates ribosomal RNA transcription in *NPM1*-mutated acute myeloid leukemia

Papaioannou et al.

Supplementary Figures

Supplementary Fig. 1. Schematic diagram of different *HOXB-AS3* transcript variants. Transcript variant is NR_033201.2 has been characterized in colorectal cancer, whereas variant NR_033202.2 is the *HOXB-AS3* transcript variant that is studied in AML. Unless otherwise stated, all experiments in the manuscript refer to transcript variant NR_033202.2. Exons are depicted as boxes and introns are depicted as lines. Strand specificity of transcripts is annotated by the direction of arrows. The protein coding *HOXB5* and *HOXB6* transcripts are also included in the diagram for reference.

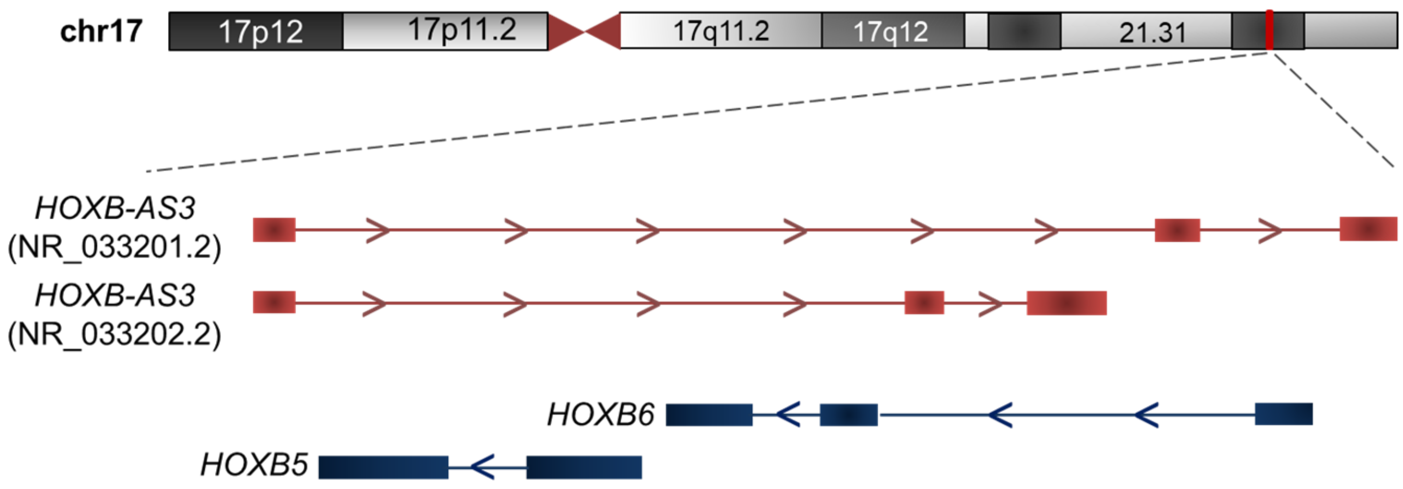

Supplementary Fig. 2. Expression profiling of the *HOXB-AS3* lncRNA in a dataset of younger adult CN-AML patients by *NPM1* mutation type. a, *HOXB-AS3* RNA expression (depicted as fold change) in younger adult CN-AML patients with wild-type *NPM1* (*NPM1*wt; n=140) and in those Type A *NPM1* mutations (*NPM1*mutA; n=208). **b,** *HOXB-AS3* RNA expression (depicted as fold change) in younger adult CN-AML patients with wild-type *NPM1* (*NPM1*wt; n=140) and in those Type B *NPM1* mutations (*NPM1*mutA; n=15). *P* values were calculated using the Wilcoxon rank sum test. . In the figures, line in boxplots indicates the mean value for each population. Whiskers indicate highest and lowest values in each population.

a

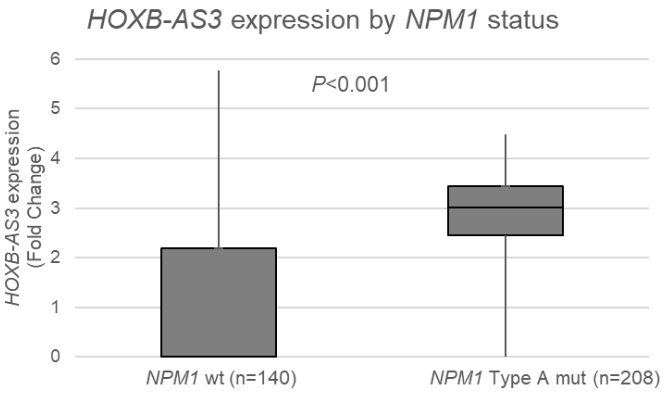

b

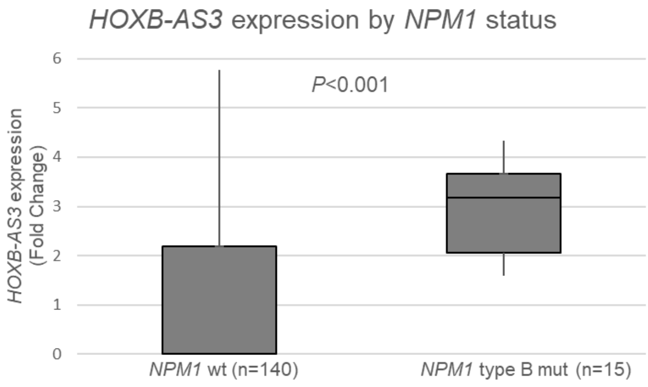

Supplementary Fig. 3. Expression of the *HOXB-AS3* lncRNA across healthy hematopoietic cells and AML subtypes. **a,** *HOXB-AS3* RNA expression among different cytogenetic subtypes of AML and ALL, in CLL, CML, MDS and in healthy hematopoietic cells isolated from bone marrow, based on the publicly available dataset of the International Microarray Innovations in Leukemia Study Group. Red color indicates high and blue low expression here and in Figs S1B and S1C. **b,** *HOXB-AS3* RNA expression among different cytogenetic AML subtypes in the publicly available dataset of The Cancer Genome Atlas project. **c,** *HOXB-AS3* expression among different molecular subtypes of cytogenetically normal AML in the publicly available dataset of The Cancer Genome Atlas project. Figs S1A-S1C were acquired through the Bloodspot portal (www.bloodspot.eu).

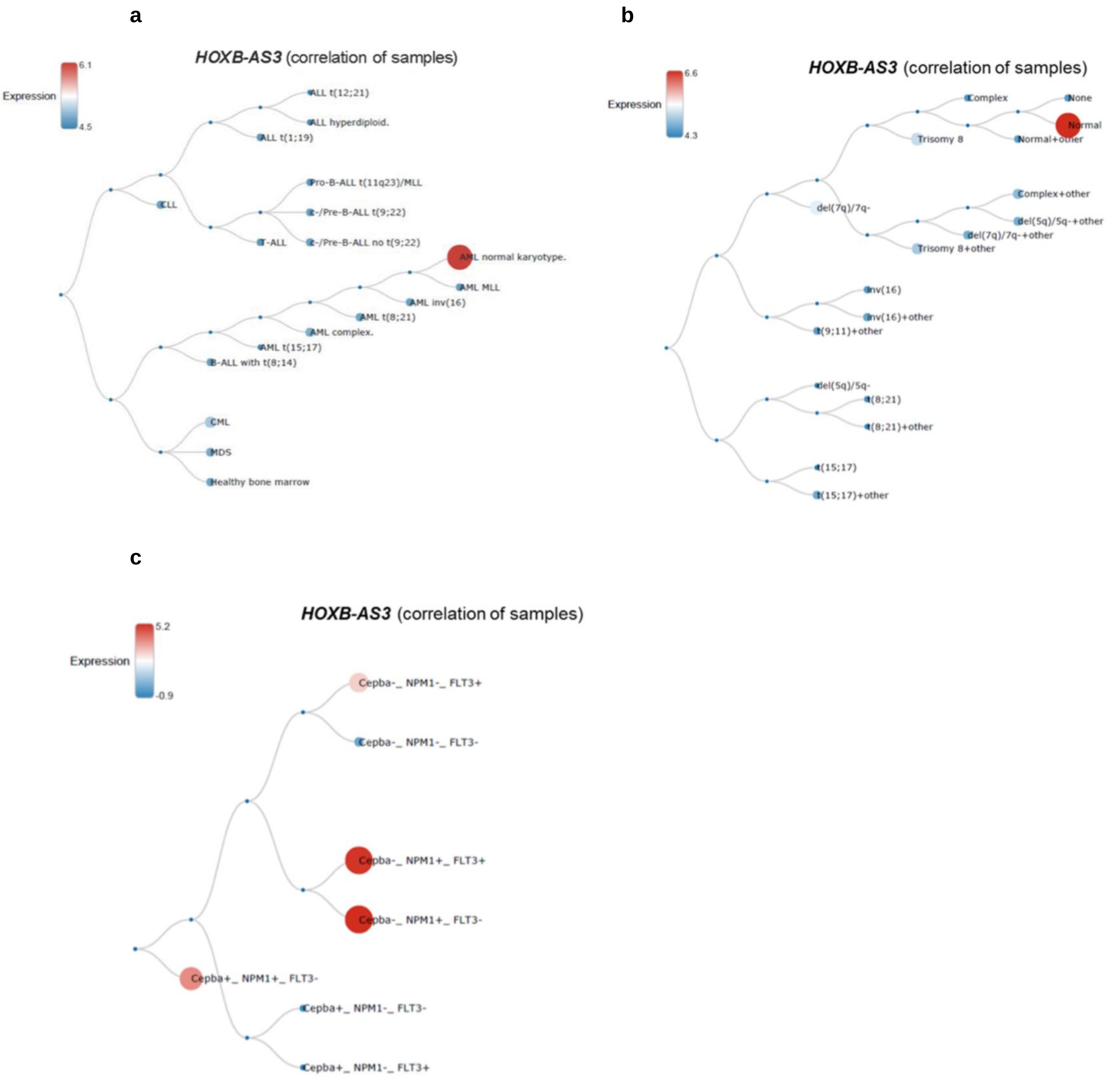
a
HOXB-AS3 (correlation of samples)
Expression
6.1
4.5
ALL t(12;21)
ALL hyperdiploid.
ALL t(1;19)
Pro-B-ALL t(11q23)/MLL
c-/Pre-B-ALL t(9;22)
c-/Pre-B-ALL no t(9;22)
CLL
T-ALL
AML normal karyotype.
AML MLL
AML inv(16)
AML t(8;21)
AML complex.
AML t(15;17)
B-ALL with t(8;14)
CML
MDS
Healthy bone marrow
b
HOXB-AS3 (correlation of samples)
Expression
6.6
4.3
Complex
None
Normal
Normal+other
Trisomy 8
del(7q)/7q-
Complex+other
del(5q)/5q-+other
del(7q)/7q-+other
Trisomy 8+other
inv(16)
inv(16)+other
t(9;11)+other
del(5q)/5q-
t(8;21)
t(8;21)+other
t(15;17)
t(15;17)+other
c
HOXB-AS3 (correlation of samples)
Expression
5.2
-0.9
Cepba-_ NPM1-_ FLT3+
Cepba-_ NPM1-_ FLT3-
Cepba-_ NPM1+_ FLT3+
Cepba-_ NPM1+_ FLT3-
Cepba+_ NPM1+_ FLT3-
Cepba+_ NPM1-_ FLT3-
Cepba+_ NPM1-_ FLT3+

Supplementary Fig. 4: Impact of *HOXB-AS3* KD on the viability of OCI-AML3 cells. **a,** Apoptosis evaluation in OCI-AML3 cells treated with either non-targeting control (scramble) or *HOXB-AS3*-targeting gapmers (*HOXB-AS3* KD). Apoptosis analysis was conducted with Annexin V and propidium iodide staining followed by flow cytometry. Results of one experiment are depicted as an example. **b,** Comparison of percentages of viable, apoptotic (early and late) and necrotic cells in in OCI-AML3 cells treated with either scramble or *HOXB-AS3* KD. Results of three independent experiments are depicted. N.S., not significant. *P* values were calculated using paired two-sided t-tests. In the figures, heights of boxplots indicate mean value with standard deviation. Whiskers indicate highest values in each population. Source data are provided as a Source Data file.

a **b**

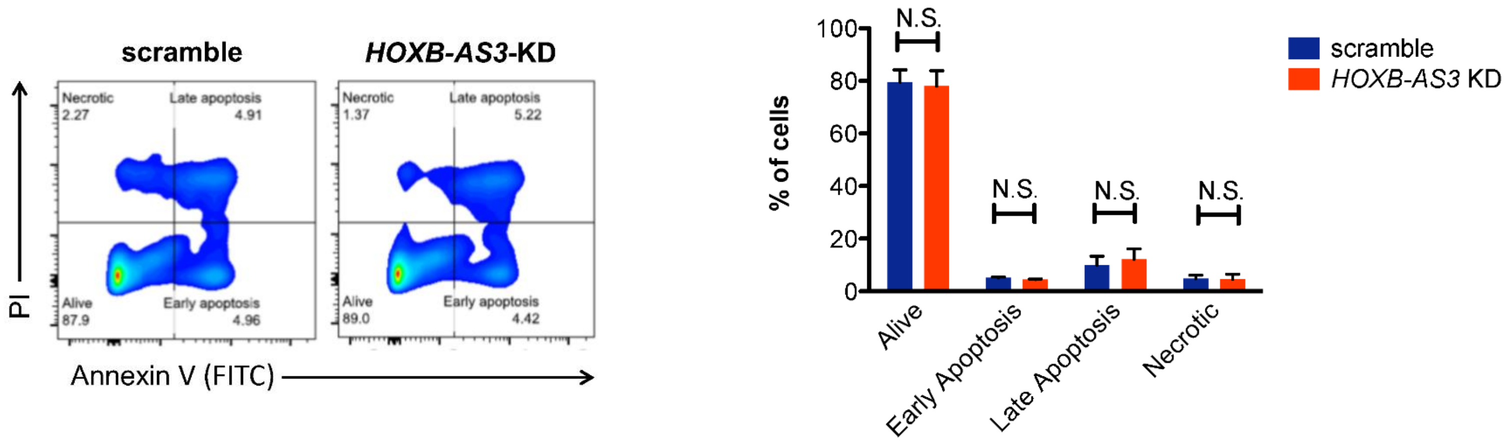

Supplementary Fig. 5: Functional significance of *HOXB-AS3* KD in AML patients with *NPM1* wild-type (*NPM1*wt) and healthy hematopoietic progenitor cells. **a** Number of colonies formed by scramble versus *HOXB-AS3* KD-treated, *NPM1*wt AML patient blasts in colony-forming unit assays. **b,** Number of total colonies formed by scramble versus *HOXB-AS3* KD-treated, CD34-selected umbilical cord blood cells, isolated from healthy donors. **c-e,** Results of **b** based on type of colony forming units: **c,** erythroid burst forming units**. d,** granulocyte, monocyte colony forming units. **e,** granulocyte, erythrocyte, monocyte, megakaryocyte colony forming units. All experiments were conducted in triplicates. N.S., not significant. *P* values were calculated using paired two-sided t-tests. In the figures, heights of boxplots indicate mean value with standard deviation. Whiskers indicate highest values in each population. Source data are provided as a Source Data file.

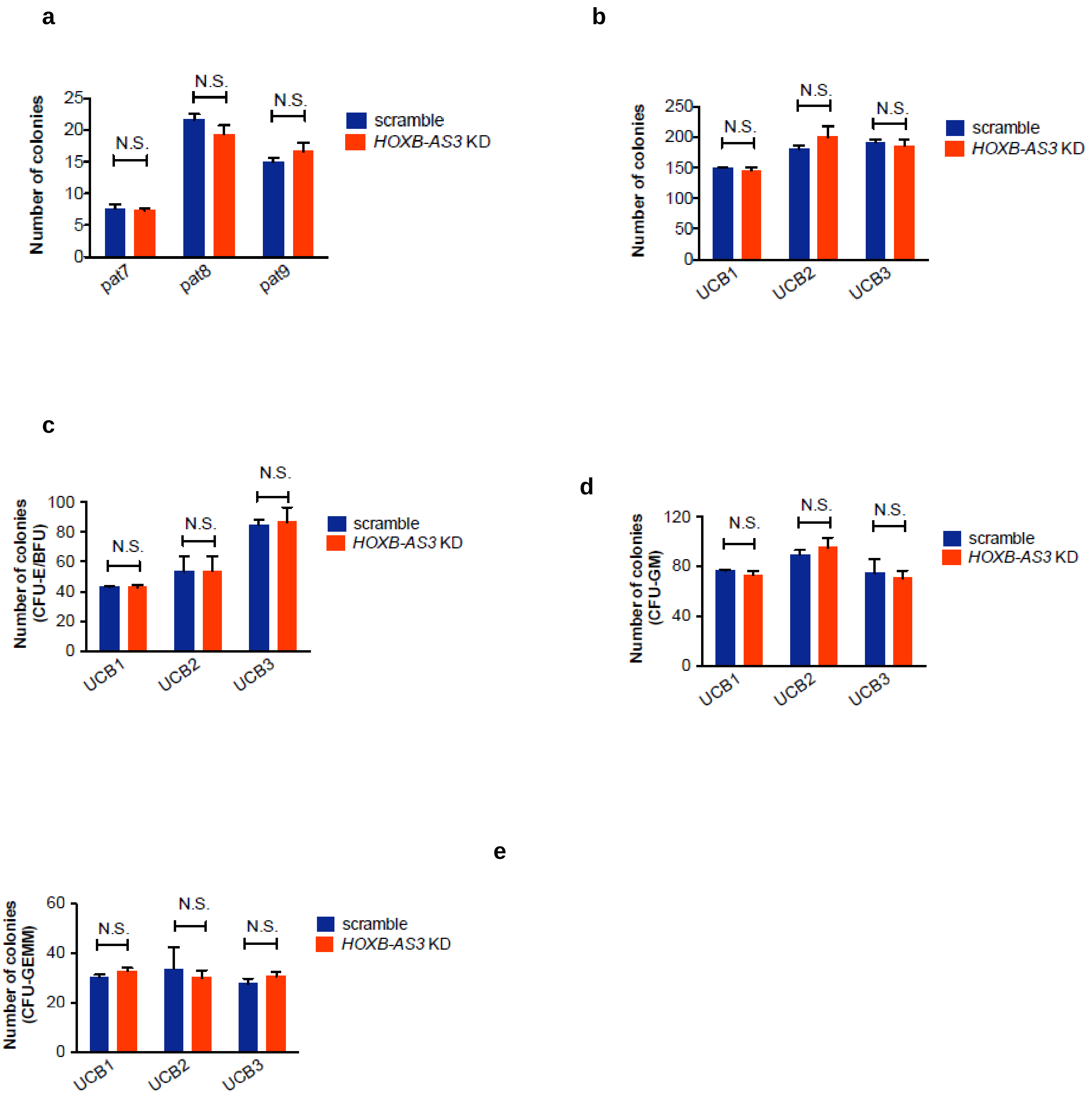
a
Number of colonies
25
20
15
10
5
0
pat7
pat8
pat9
N.S.
N.S.
N.S.
scramble
HOXB-AS3 KD
b
Number of colonies
250
200
150
100
50
0
UCB1
UCB2
UCB3
N.S.
N.S.
N.S.
scramble
HOXB-AS3 KD
c
Number of colonies (CFU-E/BFU)
100
80
60
40
20
0
UCB1
UCB2
UCB3
N.S.
N.S.
N.S.
scramble
HOXB-AS3 KD
d
Number of colonies (CFU-GM)
120
80
40
0
UCB1
UCB2
UCB3
N.S.
N.S.
N.S.
scramble
HOXB-AS3 KD
e
Number of colonies (CFU-GEMM)
60
40
20
0
UCB1
UCB2
UCB3
N.S.
N.S.
N.S.
scramble
HOXB-AS3 KD

Supplementary Fig. 6: **Interaction of the *HOXB-AS3* long-non coding RNA with the transcriptome of OCI-AML3 cells. a-b,** Expression levels of different protein-coding *HOX* genes in the four *NPM1*mut AML patient samples (scramble versus *HOXB-AS3* KD). Expression is measured as transcripts per kilobase million (tpm). With regard to the protein coding *HOX* genes, only those that were meaningfully expressed (arbitrary cut-off of >30 tpm in at least one sample) are depicted. **c-g,** Expression levels of different protein-coding *HOX* genes in the OCI-AML3 samples (scramble versus *HOXB-AS3* KD). The three time points that were evaluated (12, 24 and 48 hours) are depicted separately. Expression is measured as transcripts per kilobase million (tpm). As above, only the *HOX* genes that had expression levels >30 tpm in at least one sample are depicted. In figures A-G, blue cycles represent scramble treated and orange squares *HOXB-AS3* KD-treated blasts. **h-k,** Relative *HOXA9*, *HOXA10*, *HOXB5*, and *HOXB6* mRNA expression in scramble versus *HOXB-AS3* KD OCI-AML3 cells at 48 hours post transfection. Threshold cycle (Ct) values of *HOX* mRNA were normalized against *GAPDH* mRNA Ct values. **l,** Western blot detection of the HOXA9 protein levels in scramble and *HOXB-AS3* KD OCI-AML3 cells 48 hours post transfection. ACTB protein levels serve as loading controls between samples. *P* values were calculated using paired two-sided t-tests. In the figures, heights of boxplots indicate mean value with standard deviation. Whiskers indicate highest values in each population. Source data are provided as a Source Data file.

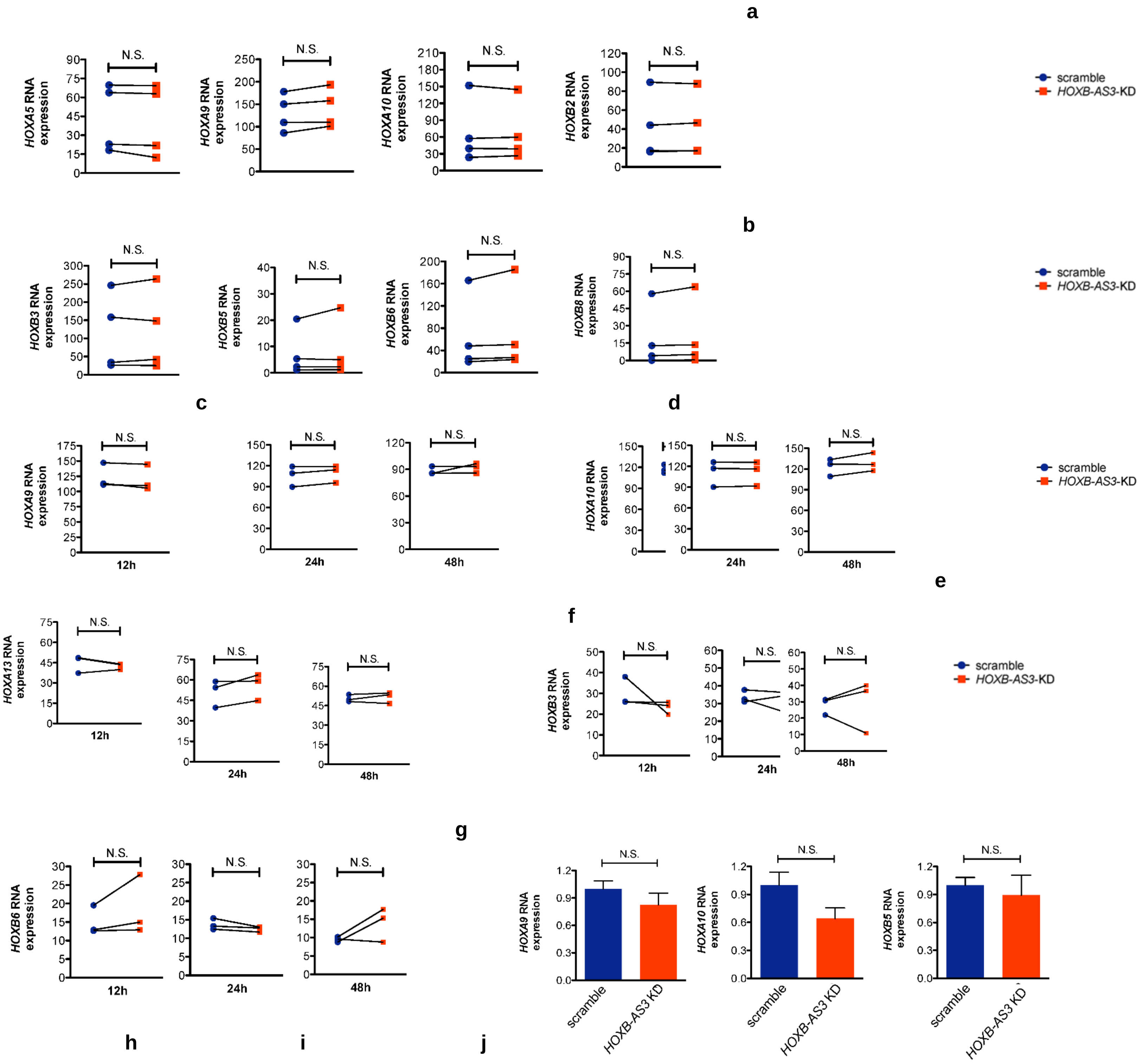
a
HOXA5 RNA expression
HOXA9 RNA expression
HOXA10 RNA expression
HOXB2 RNA expression
N.S.
scramble
HOXB-AS3-KD
b
HOXB3 RNA expression
HOXB5 RNA expression
HOXB6 RNA expression
HOXB8 RNA expression
c
d
HOXA9 RNA expression
HOXA10 RNA expression
12h
24h
48h
e
f
HOXA13 RNA expression
HOXB3 RNA expression
g
HOXB6 RNA expression
HOXA9 RNA expression
HOXA10 RNA expression
HOXB5 RNA expression
scramble
HOXB-AS3 KD
h
i
j

k

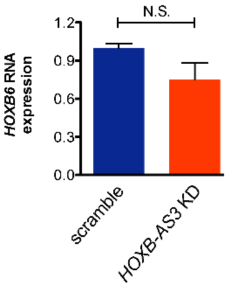
N.S.
1.2
0.9
0.6
0.3
0.0
HOXB6 RNA expression
scramble
HOXB-AS3 KD

l

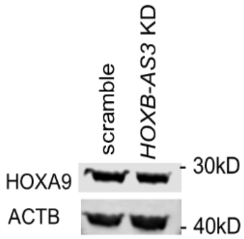
scramble
HOXB-AS3 KD
HOXA9
ACTB
30kD
40kD

Supplementary Fig. 7. Specificity of the *HOXB-AS3*-EBP1 interaction in OCI-AML3 cells. RNA-Immunoprecipitation (RIP) experiments were conducted in nuclear lysates of OCI-AML3 cells with an anti-EBP1 antibody. RNA eluates were profiled for expression levels of *HOXB-AS3* and other protein-coding (*GAPDH, ACTB*) and non-coding transcripts (*MALAT1*, *U6*). Enrichment of EBP1-immunoprecipitates is compared to IgG control. ***, $P<0.005$; N.S., not significant. *P* values were calculated using paired two-sided t-tests. In the figure, heights of boxplots indicate mean value with standard deviation. Whiskers indicate highest values in each population. Source data are provided as a Source Data file.

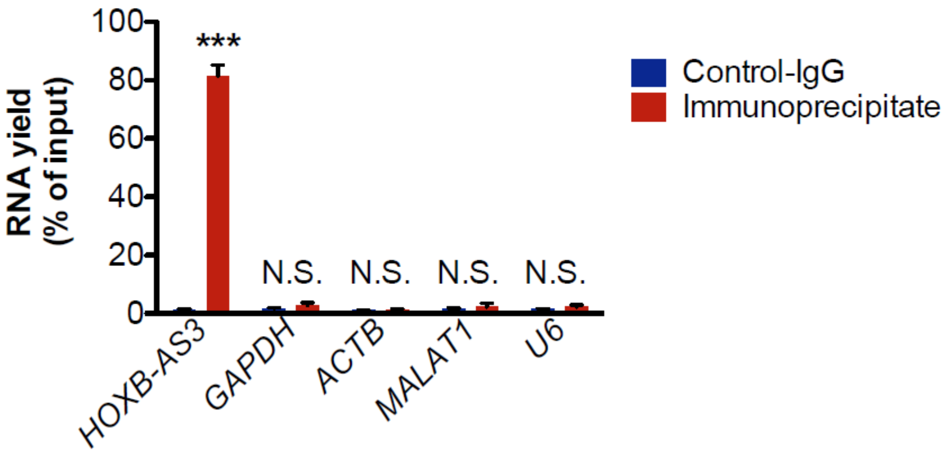

Supplementary Fig. 8. Preferential interaction of the *HOXB-AS3* lncRNA with the p48 isoform of the EBP1. RNA-Immunoprecipitation (RIP) experiments in nuclear lysates of K-562 cells, which were co-transfected with either of the FLAG epitope-tagged p42 or p48 isoforms of EBP1 and *HOXB-AS3*-overexpressing vectors. RNA eluates were profiled for expression levels of *HOXB-AS3*. Enrichment of FLAG-immunoprecipitates is compared to IgG control. *P* values were calculated using paired two-sided t-tests. In the figure, heights of boxplots indicate mean value with standard deviation. Whiskers indicate highest values in each population. Source data are provided as a Source Data file.

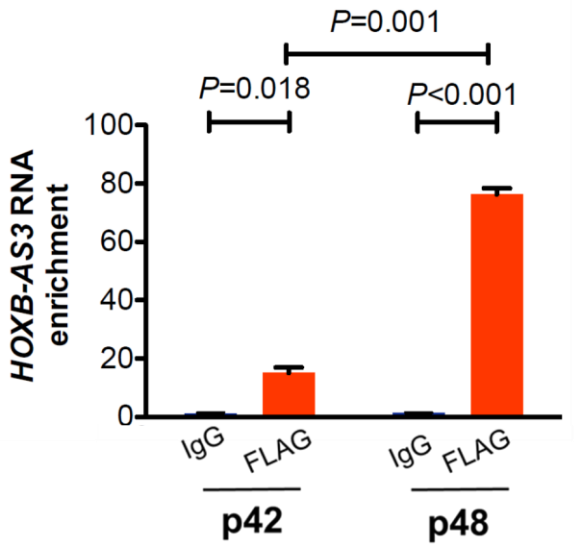

Supplementary Fig. 9. EBP1 does not interact with the mutant NPM1 protein in the nucleus of OCI-AML3 cells. a, Immunoprecipitation of the EBP1 protein in nuclear lysates of OCI-AML3 cells followed by denaturing gel electrophoresis and western blotting (WB) for the EBP1 and mutant NPM1 proteins. **b,** Immunoprecipitation of the mutant NPM1 protein in nuclear lysates of OCI-AML3 cells followed by WB for the EBP1 and mutant NPM1 proteins. Source data are provided as a Source Data file.

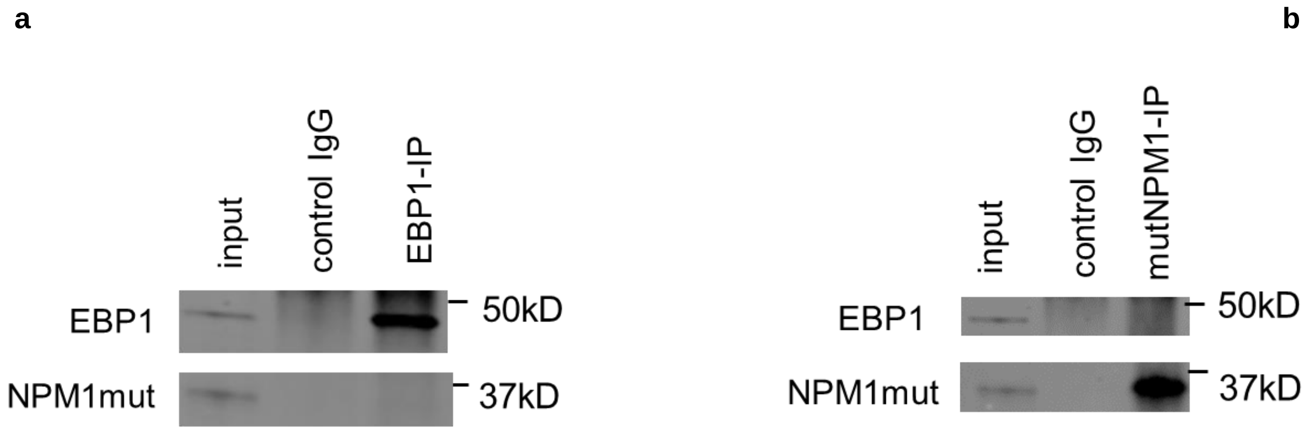

Supplementary Fig. 10. Impact of *HOXB-AS3* long non-coding RNA knock-down (KD) on the EBP1 RNA and EBP1 protein abundance. a, Relative *EBP1* mRNA and , **b,** EBP1 protein expression in OCI AML3 cells treated with scramble versus anti-*HOXB-AS3* gapmers. In Fig. b ACTB is used as loading control. *P* values were calculated using paired two-sided t-tests. In the figure, heights of boxplots indicate mean value with standard deviation. Whiskers indicate highest values of individual samples in each population. Source data are provided as a Source Data file.

a

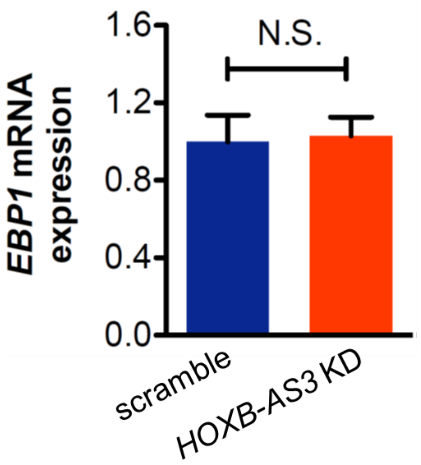

b

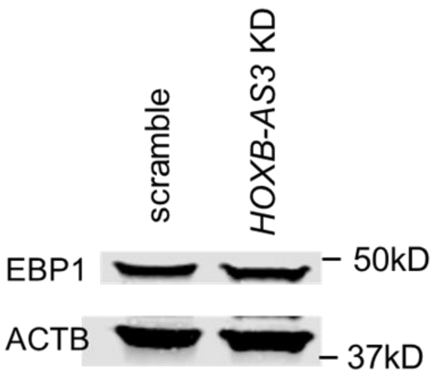

Supplementary Fig. 11. Impact of *HOXB-AS3* knock-down (KD) on the binding of EBP1 protein on non-rDNA promoters. Chromatin immunoprecipitations assays were conducted with an EBP1 targeting antibody in scramble versus *HOXB-AS3* KD treated cells followed by targeted profiling for the promoter region of *E2F1*. *P* values were calculated using paired two-sided t-tests. In the figure, heights of boxplots indicate mean value with standard deviation. Whiskers indicate highest values in each population. Source data are provided as a Source Data file.

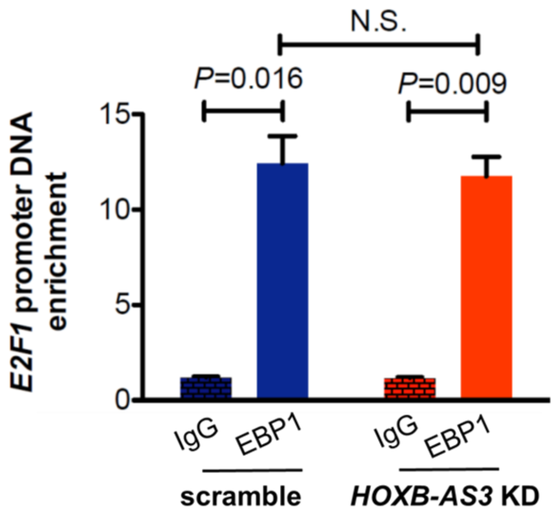

Supplementary Fig. 12. Impact of *HOXB-AS3* knock-down (KD) on the binding of NPM1 protein on the rDNA locus. Chromatin immunoprecipitations assays were conducted using NPM1-targetting antibodies in scramble versus *HOXB-AS3* KD treated cells followed by targeted profiling for the rDNA promoter region. *P* values were calculated using paired two-sided t-tests. In the figure, heights of boxplots indicate mean value with standard deviation. Whiskers indicate highest values in each population. Source data are provided as a Source Data file.

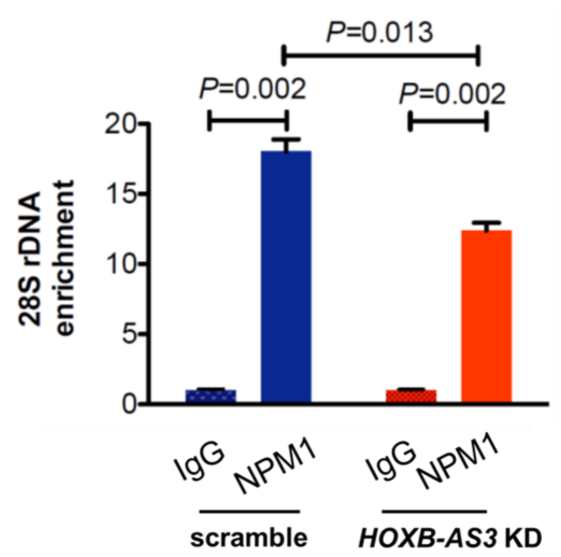

Supplementary Fig. 13. Relative *HOXB-AS3* expression in K-562 cells overexpressing *HOXB-AS3*wt or truncated *HOXB-AS3* variants 1 to 5 compared to empty vector control. ****P*<0.005; *****P*<0.001. *P* values were calculated using paired two-sided t-tests. In the figure, heights of boxplots indicate mean value with standard deviation. Whiskers indicate highest values in each population. Source data are provided as a Source Data file.

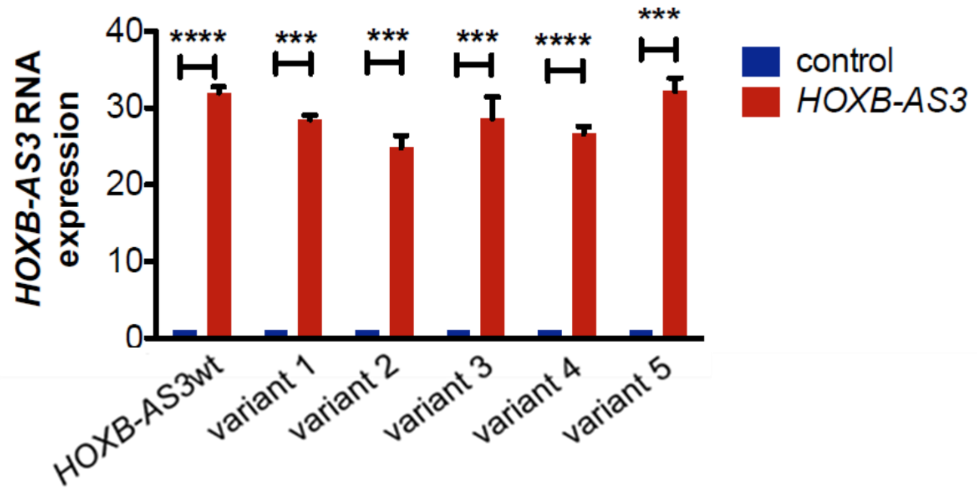

Supplementary Fig. 14. Identification of the *HOXB-AS3* regions that mediate the interaction with different *HOXB-AS3* binding proteins. a, NONO- and **b,** PHB1-targeting RIP experiments in K-562 cells overexpressing *HOXB-AS3*wt or truncated *HOXB-AS3* variants 1 to 5. The amount of *HOXB-AS3* that interacted with targeted proteins is depicted as enrichment in comparison to the rabbit IgG control. *, *P*<0.05; **, *P*<0.01; N.S., not significant. *P* values were calculated using paired two-sided t-tests. In the figure, heights of boxplots indicate mean value with standard deviation. Whiskers indicate highest values in each population. Source data are provided as a Source Data file.

a

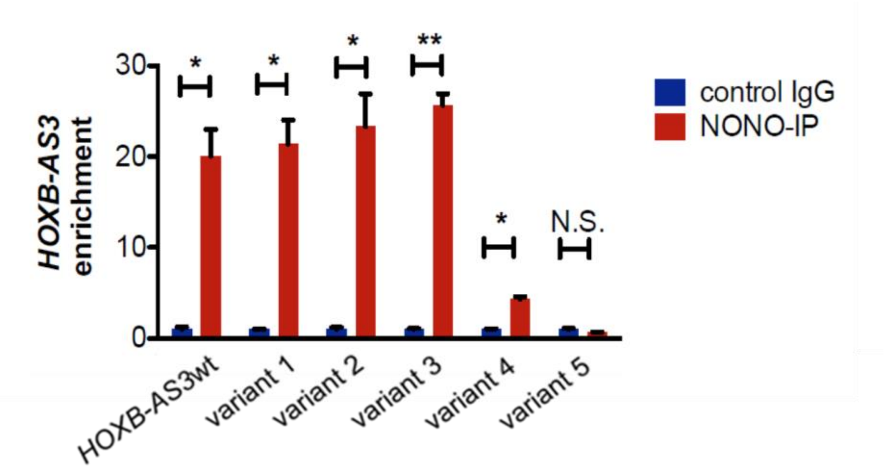

b

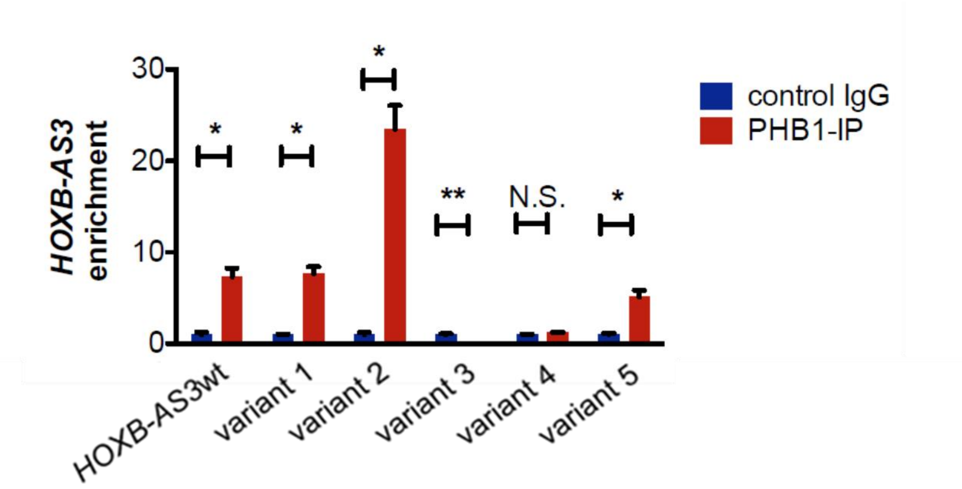

Supplementary Fig. 15. Figure exemplifying the gating strategy for the conducted flow cytometry experiments. BrdU graphs are provided as an example. **a,** Sideward (SSC-A) and forward scatter (FSC-A) settings and selection of live cells, **b, c,** negative control cells were used to set appropriate cut-offs. **b,** BrdU-negative cells were not labeled with BrdU but were stained with an anti-BrdU antibody. **c,** 7-AAD negative cells. **d,** BrdU/7-AAD-based cell cycle analysis of OCI-AML3 cells

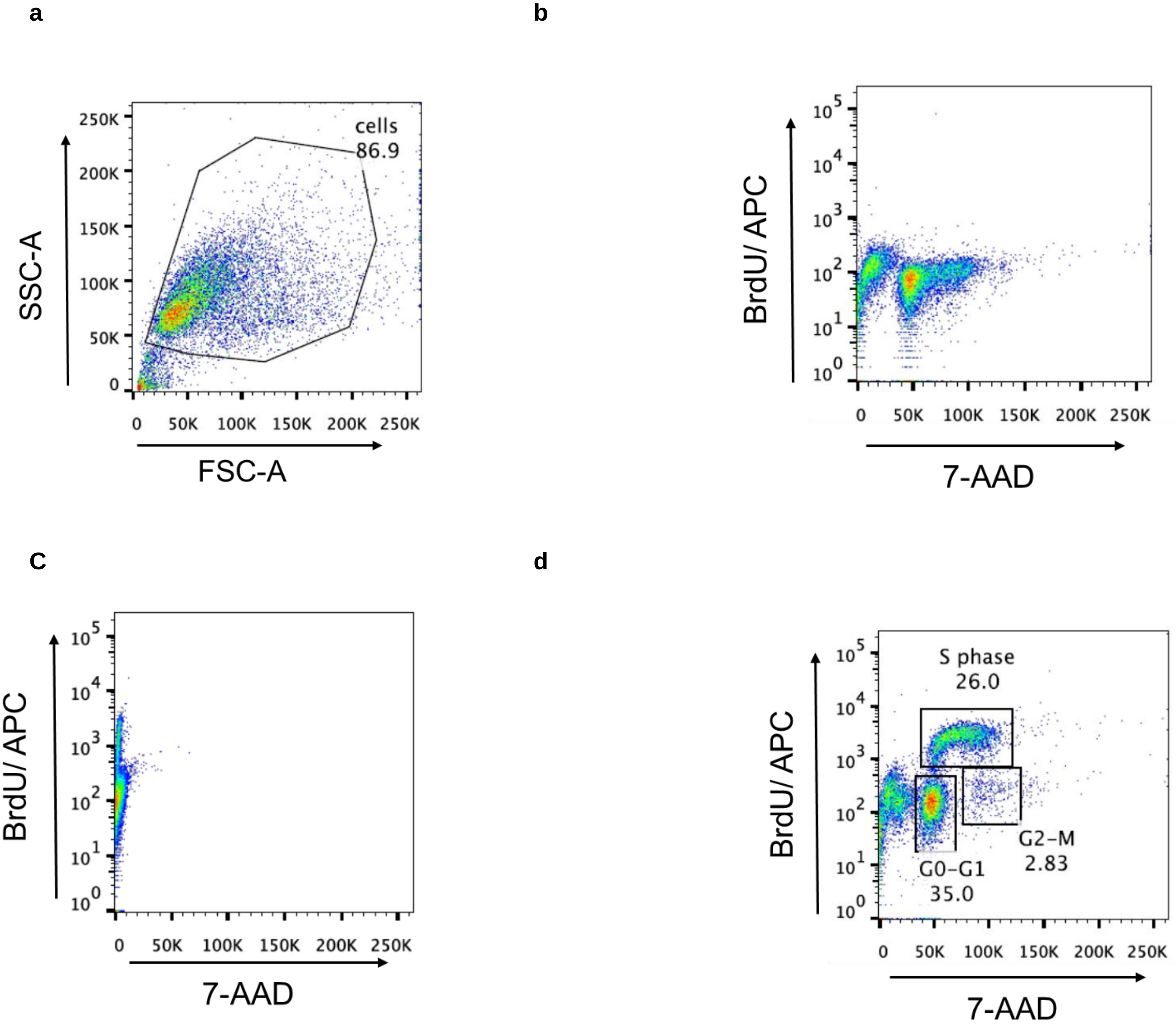

Supplementary Tables

Supplementary Table 1: List of used oligonucleotides

	**a. RT-qPCR Primers**
	HOXB-AS3
Fw_Primer	5'-CCATTCTCGATCTTTTCAAGCG-3'
Rev_Primer	5'-AGGTTGCTTGTCTGGAGATG-3'
Probe	5'-/56-FAM/CGCCTCATC/ZEN/GCTCTTATCTAAGCCC/3IABkFQ/
	Hoxb5os
Fw_Primer	5'-GAAAAGGGAGAGATGGAGGG-3'
Rev_Primer	5'-GAGCGACAGTGAGTTTACCG-3'
Probe	/56-FAM/CGTACTCAG/ZEN/GAGCAGGCCGAAC/3IABkFQ/
	**5-ETS pre-rRNA**
Fw_Primer	CCCTCGGTGAGAAAAGCC
Rv_Primer	CATAACGGAGGCAGAGACAG
Probe	/56-FAM/CGCCTCATC/ZEN/GCTCTTATCTAAGCCC/3IABkFQ/
	**b. Gapmers**
Negative Control	5'-+C*+G*+A*A*T*A*G*T*T*A*G*T*A*+G*+C*+G-3'
Anti-*NPM1*mut#1	5'-+C*+C*+A*G*A*C*A*G*A*G*A*T*C*T*T*G*A*+A*+T-3'
Anti-*NPM1*mut#2	5'-+C*+T*G*C*C*A*G*A*C*A*G*A*G*A*T*C*T*+T*+G-3'
Anti-*HOXB-AS3*#1	5'-+G*+G*A*G*G*G*A*A*T*T*G*T*A*G*+C*+G*+A-3'
Anti-*HOXB-AS3*#2	5'-+T*+G*/iMe-dC/G*T*T*G*T*A*T*T*G*G*T*A*T*G*+G*+G-3'
	**c. CRISPR gRNA sequences**
*Hoxb5os*_1a	5'-AAAAGGGAGAGATGGAGGG-3'
*Hoxb5os*_2a	5'-TGTGTGTGTGCCCCTCAGA-3'

*Hoxb5os*_2b	5'-CAGTGAGTTTACCGGGCTG-3'
*Hoxb5os*_2c	5'-GAGCGACAGTGAGTTTACC-3'
*Hoxb5os*_2d	5'-ACAGTGAGTTTACCGGGCT-3'
*Aurkb*_a	5'-GAAGAAGAGCCGTTTCATCG-3'
*Aurkb*_b	5'-GCCTGGAATACGCCCCTCGC-3'
	**d. RAP Probes**
anti-*HOXB-AS3*#1	/5Biosg/GTTGGTTAGTGGGTTTTGCCAGCTCCTTGGTTGGTGGGTCCGTGGT GAAGGAATTGAACCGAATTTTCTGGAAACTGTTGATGTCTGCAA
anti-*HOXB-AS3*#2	/5Biosg/AAAGACAAACCGGGTCTGTGTTCTCCTGGTTGGTTAGTGGGTTTTGC CAGCTCCTTGGTTGGTGGGTCCGTGGTGAAGGAATTGAACCGA
anti-*HOXB-AS3*#3	/5Biosg/CTGTTGATGTCTGCAAGTGAGTGCCGATCCCCTCCTCCATTCTCGATC TTTTCAAGCGAAGTCTACCTCTGGGCTTAGATAAGAGCGATG
anti-*HOXB-AS3*#4	/5Biosg/CGAAGTCTACCTCTGGGCTTAGATAAGAGCGATGAGGCGCTATTTTCT TTCATGCCCGACTCCAGGCGCCCAGGCCCGCCTGTAGTGGCTC
anti-*HOXB-AS3*#5	/5Biosg/GGTGAGGAGGAAGCCGAGGGTCGTGGAGGCTCTACTTGGCCCTCCTT TCCCTTCACCCCTTGACTTTGTCTCCCTTTGTCGATAGCAAAC
anti-*HOXB-AS3*#6	/5Biosg/TTTATTTTACTTTCTTTGTCTTAATTTATCTCGTTCTAAATTGATACAGAC GACCCAAATAAAACTTTAAAATAGGGGGTGGGGAAGGGA
anti-*HOXB-AS3*#7	/5Biosg/TGAGTCCGGGAGCAAGATCCTAAGAGGTGCGAGTTTACCAGGCCGGT GTGGCCGCATCCCGGCCGCTCTCGGAGCCTGACCACGCCACGC
anti-*HOXB-AS3*#8	/5Biosg/CCGGGGCTTCCTCCGAGCCTCTCTTCCCGCTCTCTGCGCCGCCGCCG GCAGCCTGGAACCTTCTCCGCTGGTGCGGATATCGCTGGGTTC
anti-*HOXB-AS3*#9	/5Biosg/GTGCGGATATCGCTGGGTTCCCGGCAGCACTGGCATGTGAGGCGGTG GCGCTAAGGGACGTCCTGGTTTCTATAGGGCCTGGAATCTCCG
anti-*U1*#1	/5Biosg/CAGGGGAAAGCGCGAACGCAGTCCCCCACTACCACAAATTATGCAGT CGAGTTTCCCACATTTGGGGAAATCGCAGGGGTCAGCACATCC
anti-*U1*#2	/5Biosg/TTATGCAGTCGAGTTTCCCACATTTGGGGAAATCGCAGGGGTCAGCA CATCCGGAGTGCAATGGATAAGCCTCGCCCTGGGAAAACCACC
	**e. Overlap PCR Primers**
1st Primer_Fw	5'-GGATAGTTTGCTATCGACAAAGGGAGAC-3'
1st	5'-GTAGTGGCTCGTGAGGAGGAAGC-3'

Primer_Rev	
2nd Primer_Fw	5’-CCACTACTCGGCACTCACTTGCAG-3’
2nd Primer_Rev	5’-GAGTGCCGAGTAGTGGCTCCATC-3’
3rd Primer_Fw	5’-ACTTGCAGAACCAGGAGAACACAGAC-3’
3rd Primer_Rev	5’-CTGGTTCTGCAAGTGAGTGCCGA-3’
Alt 5 end_Fw	5’-CGGCTTCCTCCTCACCAGC-3’
Alt 3 end_Rev	5’-CCGTCAGTTCCACTCGGTTGT-3’

Abbreviations: Fw, forward; Rev: reverse; FAM, 6-carboxyfluorescein; ZEN, ZEN internal quencher; 3IABkFQ, 3’ end Iowa black quencher; Biosg, biotinylated DNA base
+, locked-nucleic acid-modified DNA base; *, phosphorothioated DNA.

Supplementary Table 2. Cytogenetic and gene mutational profiles of the 12 AML patients, whose samples were used for *in vivo* and *in vitro* experiments

Patient	Karyotype	Genes Mutated
**pat1**	46,XY[2]	***NPM1*, *TET2*, *IDH1***
**pat2**	46,XY[5]	***NPM1*, *FLT3*-ITD, *TET2***
**pat3**	46,XX[20]	***NPM1*, *DNMT3A***
**pat4**	46,XY[6]	***NPM1*, *FLT3*-ITD, *IDH1***
**pat5**	46,XX[20]	***NPM1*, *FLT3*-ITD**
**pat6**	46,XX[7]	***NPM1*, *FLT3*-ITD, *TET2***
**pat7**	47,XY,+5[cp5]/46,XY[2]	***TET2***
**pat8**	45,XY,psu dic(5;17)(p15;p13)[11]/ 46,XY,t(2;6)(q13;p25)[5]/46,XY[4]	***RUNX1***
**pat9**	46,XX[20]	***FLT3*-TKD, *ASXL1*, *DNMT3A***
**pat10**	46,XX,inv(16)(p13q22)[20]	***FLT3*-TKD**
**pat11**	46,XX[20]	***FLT3*-ITD, *RUNX1*, *IDH1***
**pat12**	42-46,XY,add(2)(p13),del(5)(q13q31),del(9) (q13q22),der(11)t(11;11)(p15;q13) add(11)(q25), dup(11)(q14q23),del(12)(p13),-13,ins(14;?) (q24;?),-17,add(19)(p13.3),add(21)(p11.2)[cp8]/ 41-46,sl,+mar[cp12].ish del(5)(EGR1-), der(11)t(11;11)add(11)(MLL++),dup(11)(MLL++)	

Abbreviations: *FLT3*-ITD, internal tandem duplications of the *FLT3* gene; *FLT3*-TKD, tyrosine kinase domain mutations of the *FLT3* gene

Supplementary Table 3. List of candidate RNA binding proteins identified to interact with the *HOXB-AS3* or the *U1* transcripts by mass spectrometry. The mean number of identified peptides for each candidate interacting protein is also provided. Proteins previously reported to interact with *U1* RNA are highlighted in yellow.

Putative *HOXB-AS3* interactors	Number of identified peptides	Putative *U1* interactors	Number of identified peptides
EBP1	13	RU17	20
DHX9	9	SNRPA	12
PHB1	9	RUXE	8
MATR3	8	SMD2	7
NONO	8	SMD3	7
HMGB2	7	PLEC	5
HNRNPU	6	DDX3X	4
PRDX3	6	FLNB	4
SFPQ	6	PPIB	3
PARK7	5		
PTMA	5		
SRSF2	5		
XRCC6	5		
HNRNPH1	5		
SF3B3	4		
HNRNPC	4		
HNRNPM	4		
HNRNPK	4		
HNRNPF	4		
STIP1	4		
PCBP1	3		
TCEA1	3		

Supplementary Table 4. List of used antibodies

Antibody	Provider	Identifier
Mouse Monoclonal anti-NPM1	Abcam	Cat# ab10530
Rabbit Polyclonal anti-EBP1	Abcam	Cat# ab33613
Mouse Monoclonal anti-ACTB	Cell Signaling Technology	Cat #58169
Rabbit Polyclonal anti-DHX9	Abcam	Cat# ab26271
Rabbit Monoclonal anti-HOXA9	Abcam	Cat# ab140631
Rabbit Polyclonal anti-SC35 (SRSF2)	Abcam	Cat# ab28428
Rabbit Polyclonal anti-nmt55/p54nrb (NONO)	Abcam	Cat# ab70335
Rabbit Polyclonal anti-NPM1 mutant	OriGene	Cat# TA301725
Mouse Polyclonal anti-PHB1	Cell Signaling Technology	Cat# 2426S
Rabbit Monoclonal anti-POLR1A	Cell Signaling Technology	Cat# 24799
Mouse Monoclonal anti-MATR3	Millipore Sigma	Cat# MABN1587
Normal Rabbit IgG	Millipore Sigma	Cat# 12-370
Normal Mouse IgG	Millipore Sigma	Cat# 12-371
Mouse Monoclonal Anti-Mouse CD45.1, APC Conjugated	BD Biosciences	Cat# 558701
Mouse Monoclonal Anti-Human CD45, Phycoerythrin Conjugated	BD Biosciences	Cat# 555483
Goat anti-Rabbit IgG, IRDye 800CW Conjugated	LI-COR Biosciences	Cat# 925-32211
Goat anti-Mouse IgG, IRDye 680CW Conjugated	LI-COR Biosciences	Cat# 925-68070
Goat anti-Mouse IgG, Alexa Fluor 488 Conjugated	Thermo Fisher Scientific	Cat# A-11029
Goat anti-Rabbit IgG, Alexa Fluor 488 Conjugated	Cell Signaling Technology	Cat# 4412
Rabbit anti-FLAG	Sigma-Aldrich	Cat# F7425
